# Modulation of the association between blood glucose homeostasis and social hierarchy among co-housed mice by diet and amygdala activities

**DOI:** 10.1016/j.jphyss.2026.100058

**Published:** 2026-01-20

**Authors:** Rikako Ukichi, Yukari Takahashi, Momoyo Ibukuro, Yae K. Sugimura, Keiichiro Matoba, Rimei Nishimura, Fusao Kato

**Affiliations:** a Department of Neuroscience, Japan; b Division of Diabetes, Metabolism and Endocrinology, Department of Internal Medicine, The Jikei University School of Medicine, Minato-ku, Tokyo 105-8461, Japan

**Keywords:** Diabetes mellitus, Social hierarchy, Tube test, Chemogenetics, Glucose metabolism, Basolateral amygdala

## Abstract

Recent clinical studies suggest that individual psychosocial traits play a significant role in the onset and progression of diabetes. To examine whether glucose homeostasis depends on the social rank of individual mice, we analyzed the effects of dietary fat content on the hierarchy formed among co-housed mice and evaluated how perturbing rank by inhibiting amygdala neuronal activity influences glucose regulation. Social rank among four co-housed mice was assessed using the tube test. Switching to a high-fat diet altered blood glucose homeostasis, particularly by affecting rapid responses, and disrupted the established hierarchy, with the degree of disruption varying according to each mouse’s rank. In contrast, chemogenetic inhibition of neuronal activities in the basolateral amygdala and surrounding area in the lowest-ranking mice modified both glucose homeostasis and its association with social rank. These findings provide mechanistic insight into the interaction between glucose regulation and psychosocial status.

## Highlights

• Glucose metabolism traits of co-caged mice were associated with individual social rank.
• Dietary fat content changes disrupted established social rank and glucose metabolism.
• Rapid glucose responses and pancreatic islet size depended on individual social rank.
• Rapid glucose responses were associated with the social rank of individual mice.
• Inhibiting amygdala neurons in subordinate mice altered cage-mates’ glucose metabolism.

## Graphical Abstract

**Figure:**
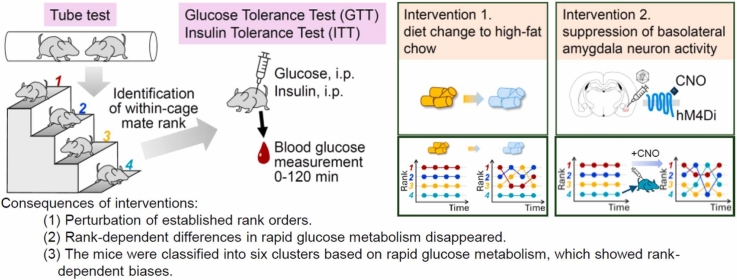

## Introduction

Diabetes mellitus affects approximately 10 % of the global adult population and represents a growing public health burden due to its chronic nature and associated complications [1], [2]. The maintenance and optimization of blood glucose levels is essential for the proper functioning of widespread organs, including the brain. In addition to the local autonomous feedback mechanisms within pancreatic β-cells underlying glucose-induced insulin release [3], the central nervous system plays an indispensable role in blood glucose regulation, and its dysregulation is also implicated in the pathogenesis of type 2 diabetes [4]. For example, parasympathetic efferent activity and hypothalamic regulatory circuits are critically involved in glucose metabolism [5], [6], [7], [8]. Moreover, these brainstem and autonomic circuits are modulated by higher-order networks such as limbic and cortical systems. Indeed, psychosocial status and individual behavioral traits have been reported to be associated with susceptibility to type 2 diabetes [9], [10], [11], [12].

In contrast, sustained elevations in blood glucose, such as those caused by excessive food intake, can modify brain network activity through multiple mechanisms. For example, consumption of a high-fat diet for more than 4 weeks induces anxiolytic-like behaviors accompanied by increased levels of mature brain-derived neurotrophic factor (mBDNF) in the medial prefrontal cortex (mPFC), a brain region strongly linked to psychosocial traits and behavioral tendencies [13]. Even short-term dietary manipulations exert marked effects in rats, consumption of a high-fat diet for only 3–5 days enhances excitatory synaptic transmission in the dorsal motor nucleus of the vagus nerve, a key site linking brain activity and parasympathetic outflow [14]. Remarkably, a single 24-h exposure to a high-fat diet upregulates inflammatory mediators and immune cells in the gut and nodose ganglion as well as the hypothalamus in mice [15]. These findings suggest that changes in diet modulate glucose metabolism not only through local pancreatic mechanisms, as demonstrated in many studies [16], [17], but also by altering brain functions that influence autonomic optimization of the on-site regulation as well as psychosocial status and behavioral traits, which in turn affect the neural regulation of glucose metabolism.

The present study was undertaken to address key questions regarding the interaction between individual behavioral traits and blood glucose regulation. To assess the psychosocial traits, we measured the hierarchical rank of individual mice among co-housed mice as a proxy. For this purpose, we used a recently established, reliable, and non-invasive method called the “tube test” [18], [19], [20], [21] and analyzed the effects of two interventions—(1) a change in dietary fat, and (2) the chemogenetic suppression of basolateral amygdala (BLA) neuronal activity, which underlies the expression of submissive behaviors—on glucose metabolism in mice with an identified hierarchical rank. Glucose metabolism was evaluated with a conventional glucose tolerance test (GTT) and insulin tolerance test (ITT) [22]. With these interventions, we asked: (1) how dietary changes, which affect glucose metabolism traits [23], [24], influence established social hierarchy; (2) how the hierarchical rank of individual mice affects the glucose metabolism change following dietary modification; and (3) whether experimental perturbation of psychosocial hierarchy by manipulating BLA activity alters the hierarchical rank and glucose metabolism of the cohoused mice.

## Methods

### Animal experiment ethics

Animal handling was approved by the Institutional Animal Care and Use Committee of Jikei University (Nos. 2018–017, 2019–001, 2019–007) and conformed to the Guidelines for Proper Conduct of Animal Experiments of the Science Council of Japan [25] and the guidelines of the International Association for the Study of Pain [26].

### Animal housing and maintenance

**Fig. 1:**
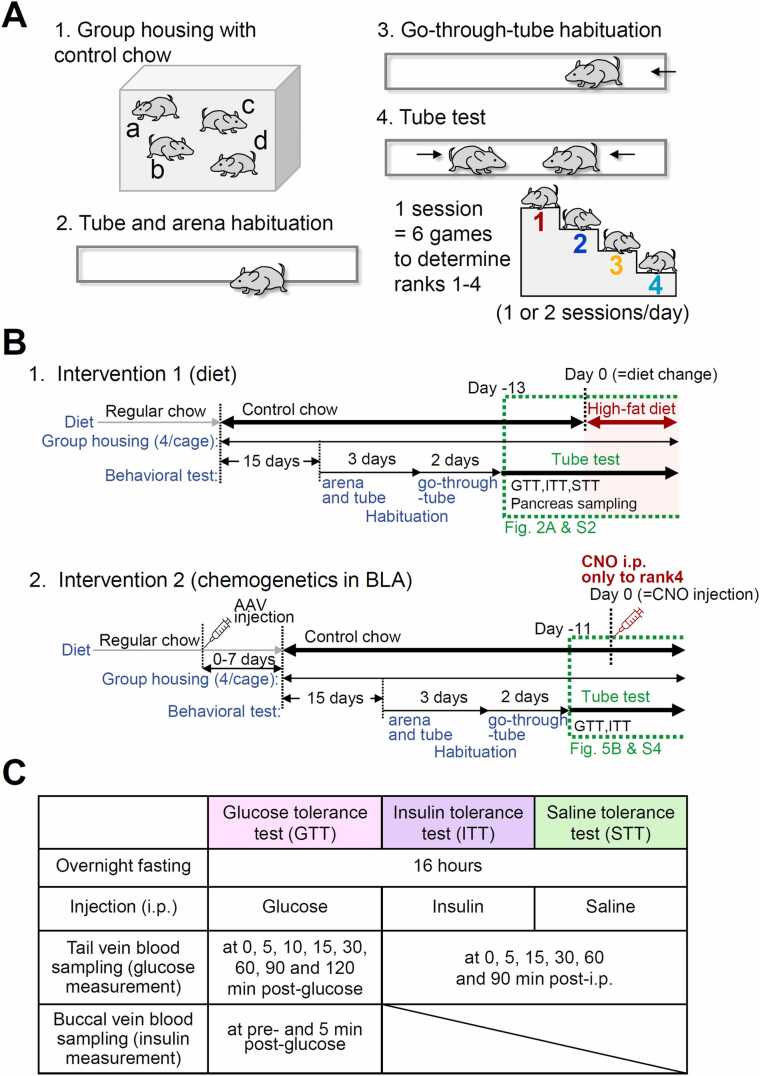
Experimental protocol for the assessment of social rank and glucose metabolism. **A.** Protocol for rank assessment using the tube test. Male C57BL/6 J mice were housed in groups of four per cage (a–d in A1). Mice first underwent habituation in the behavioral testing arena (“tube-and-arena habituation”, A2), followed by training to pass through a transparent acrylic tube (“go-through-tube habituation”, A3). Social rank within each cage was determined by a round-robin tube test consisting of six pairwise matches/session (A4; see also Figure S-1A). Ranks of four cage mates were assigned from 1 (most dominant) to 4 (least dominant). **B.** Experimental timelines for dietary and chemogenetic interventions. **Intervention 1 (diet)**. Mice were group-housed and maintained on regular chow for 3 days, after which the chow was replaced with control chow until Day 0. Following the “arena” (3 days) and “go-through-tube habituation” (2 days), tube tests began on Day −13. The chow was switched to a high-fat diet on Day 0. **Intervention 2 (chemogenetics)**. Mice were maintained on regular chow before injection of AAV solution into the BLA. After 0–7 days of recovery, the chow was replaced with control chow for the remainder of the experiment. Mice were then group-housed for 15 days to allow sufficient expression of the exogenous genes. Following the arena” (3 days) and “go-through-tube habituation” (2 days), tube tests began on Day −11. Rank_pre-CNO_ was determined on Day −7 before pre-CNO GTT and ITT. On Day 0, rank_pre-CNO_4 mice received CNO (intraperitoneal). The tube test and metabolic assessments (surrounded by green broken lines) are described in greater detail in each figure legend (Figs. 2-A, S2, 5-B, and S4). **C.** Protocol for metabolic assessments. GTTs, ITTs, and STTs were performed after 16 h fasting according to the protocol summarized in this table.

C57BL/6 J male mice (4 weeks old) were purchased from SLC Japan and housed in groups (four mice/cage) in isolated ventilated cages with free access to food and water (Fig. 1-A1 and Fig. 1-B). Four mice to be co-housed were chosen randomly and placed in home cages located in a temperature/humidity-controlled room with a light/dark cycle (07:00–19:00, white light; 19:00–00:00, red light; 00:00–07:00, dark). The floors of these home cages were covered with conventional soft animal floor bedding (Alpha-dri; EP Trading, Tokyo, Japan). First, the mice were fed with control chow (see Table 1 for its composition) from the day of group housing. Body weight was measured a few days after the start of group housing (details provided in the Results section). We used a total of 100 mice (48 mice for the intervention 1 experiment and 52 mice for the intervention 2 experiment), which are statistically minimally sufficient to draw meaningful conclusions for each of the tests.

**Table 1:** Composition of the diet.

(1) Control chow (D12450J)						
Class description	Ingredients	Weight (g)	Macros			
			g	g%	kCa	kCa%
Protein	Casein, Lactic, 30 Mesh	200.00	203	19	812	20
	Cystine, L	3.00				
Carbohydrate	Starch, Corn	506.20	704	67	2816	70
	Lodex 10	125.00				
	Sucrose, Fine Granulated	72.80				
Fiber	Solka Floc, FCC200	50.00				
Fat	Soybean Oil, USP	25.00	45	4	405	10
	Lard	20.00				
Mineral	S10026B	50.00				
Vitamin	Choline Bitartrate	2.00				
	V10001C	1.00				
Dye	Dye, Yellow FD&C #5, Alum. Lake 35–42 %	0.04				
	Dye, Blue FD&C #1, Alum. Lake 35–42 %	0.01				
Total:		1055.05		90	4033	100

(2) High-fat diet (D12492)						
Protein	Casein, Lactic, 30 Mesh	200.00	203	26	812	20
	Cystine, L	3.00				
Carbohydrate	Lodex 10	125.00	197.8	26	791.2	20
	Sucrose, Fine Granulated	72.80				
Fiber	Solka Floc, FCC200	50.00				
Fat	Soybean Oil, USP	25.00	270	35	2430	60
	Lard	245.00				
Mineral	S10026B	50.00				
Vitamin	Choline Bitartrate	2.00				
	V10001C	1.00				
Dye	Dye, Blue FD&C #1, Alum. Lake 35–42 %	0.05				
Total:		773.85		87	4033.2	100

### Assessment of social rank in mice

We used the tube test [18] to assess the rank of the mice in each cage. Before the tube test, the mice were habituated to the experimental setups using the following four steps.

#### Pre-test handling and tube test habituation

After the beginning of the group housing (Figs. 1-A and 1-B), the mice were handled for a few minutes daily to habituate them to the experimenter. After 15 days of this handling-only period, each mouse was placed in a rectangular observation area (∼45 cm × ∼30 cm, surrounded by walls ∼30 cm high, called the “arena”) with a transparent acrylic tube (30 cm in length and 3 cm in diameter) fixed to the center of the floor for ∼5 min (Fig. 1-A2). This tube-and-arena habituation was performed once a day for 3 days. This was followed by “go-through-tube” habituation (Fig. 1-A3), where a single mouse was placed at either edge of a tube and allowed to enter and walk through the tube. This procedure was repeated three times in each direction. This “go-through-tube” habituation was performed once a day for 2 days (Fig. 1-B).

#### Tube test games and sessions for rank determination of each mouse

**Fig. 2:**
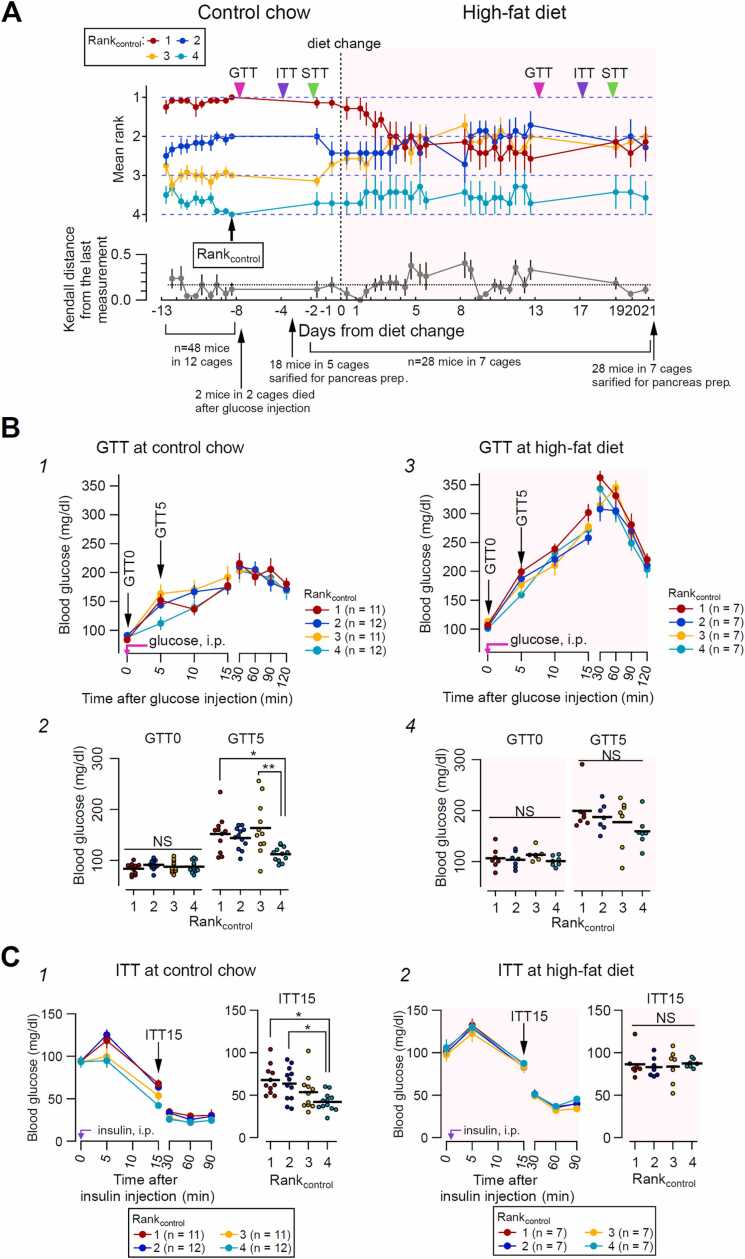
Effect of a dietary intervention on glucose metabolism. **A.** Upper panel, rank over time. Y-axis: mean rank (n = 48 mice, 4 ranks from 12 cages, mean ± SEM). X-axis: days relative to diet change (Day 0). Line colors represent the baseline rank (rank_control_). Colored lines and symbols represent the means of the ranks of mice assigned to each rank_control_ at each assessment (mean ± SEM of 12 cages). Control chow was switched to a high-fat diet (HFD) on Day 0. Pink, purple, and green arrowheads show GTT, ITT, and STT timings, respectively. Lower Y-axis: mean Kendall distance between consecutive tube-test sessions for all mice (mean ± SEM). The horizontal broken line indicates 0.17. This value corresponds to the smallest non-zero Kendall distance for a cage assuming linear pattern (see Fig. S1-B1). **B.** Time course of blood glucose concentrations in GTTs before (B1, B2) and after (B3, B4) a diet change for different rank_control_ mice. B1 and B3 indicate glucose concentrations over time; colored lines and symbols represent the mean of the mice belonging to the same rank_control_ (mean ± SEM); B2 and B4 indicate the glucose concentration at 0 and 5 min after glucose injection (GTT0 and GTT5, respectively). Horizontal bars indicate group means. B1 and B3, n = 11–12; B2 and B4, n = 7; *p < 0.05; **p < 0.01; NS, not significant (one-way ANOVA with Tukey’s post-hoc test). The glucose concentration in the blood sampled from mice of different rank_control_ did not significantly differ at any point from GTT10 to GTT120 (one-way ANOVA). **C.** Blood glucose concentrations in ITTs with control chow (C1) or HFD (C2). Data are mean ± SEM. Left panels of C1 and C2 indicate the postinjection time course. ITT15 indicates the blood glucose concentration 15 min after insulin injection. Right panels of C1 and C2 indicate the mean values by rank_control_ (horizontal bars) and individual values (colored filled circles) of the ITT15 pre- (C1) and post- (C2) diet change. *p < 0.05; NS, not significant (one-way ANOVA with Tukey’s post hoc test). The glucose concentration in the blood sampled from mice of different rank_control_ did not significantly differ at any point, except at ITT15 (one-way ANOVA).

After habituation, two mice were taken from the cage, and the experimenter placed each mouse at one end of the tube by holding its tail (Fig. 1-A4). At the start of the test, both tails were released simultaneously, allowing the mice to walk forward and meet in the middle of the tube. The mouse that retreated from the tube (usually by walking back) before the opponent was designated the “loser” and the mouse that stayed inside the tube was the “winner”. These winners and losers were determined within 1 min of observation. After the winner and loser were determined, the mice were immediately returned to the co-housing cage, and the tube was cleaned with 70 % ethanol. A “session” consisted of a total of six "games" between every pair from four mice in a cage (_4_C_2_ = 6) to determine the rank of each mouse (Figure S1-A). Figure S1-A indicates how the rank of each mouse from a cage was determined according to the win-and-lose patterns in one session (table in Figure S1-A, right). Of all 569 tube test session performed in the experiments reported in the present study, linear patterns, non-linear patterns and circular patterns were observed in 527, 35 (20 and 15 for "1 top" and "1 bottom" pattern, respectively), and 7 sessions, respectively (Figure S1-B). The rank on the fifth day of the tube test of all mice was assigned to rank_control_ (intervention 1, Fig. 2-A) or rank_pre-CNO_ (intervention 2, Fig. 5-B1) and used to represent the social hierarchy of the mouse of interest. All games were video-recorded via a web camera (Logicool HD Webcam C525; Logitech, Lausanne, Switzerland). We placed an illuminated plate (∼35 cm × 47 cm, A3–500-W; Trytec, Oita, Japan) below the tube, and the light was fixed at ∼200 lux at the level of the tube. The tube test was performed twice a day (at approximately 14:00–16:00 and 19:00–21:00).

#### Kendall distance

To evaluate the stability of the existing hierarchy over consecutive matches, the Kendall distance (KD) was calculated between two subsequent sets of rank patterns measured in two consecutive tube tests for four mice in each cage [27] (Figure S1-B). The number of adjacent swaps required to make two different rank orders composed of 4 values identical were calculated and divided by 7 (i.e., the number of adjacent swaps required to make "1–2–3–4" to "4–3–2–1"). KD provides information about how different the two sets of rank orders are (0, identical; 1, most different).

### Assessment of glucose metabolism

#### Glucose tolerance tests

Intraperitoneal GTTs were performed as described previously [22]. Briefly, the mice were fasted overnight (16 h) with free access to water. The mice were injected intraperitoneally with 1 g of glucose per kilogram of body weight (5 % glucose solution). Venous blood was obtained from the tail vein at 0, 5, 10, 15, 30, 60, 90, and 120 min after injection and measured for glucose using an automatic glucometer (OneTouch Verio™ IQ meter; LifeScan, Inc., Milpitas, CA / Medisafe FIT; Terumo Corporation, Tokyo, Japan). The timings immediately before glucose injection and 5 min post-injection were designated “GTT0” and “GTT5”, respectively. The difference in glucose values between those sampled at GTT0 and GTT5 was referred to as “Δglucose”, indicating changes in glucose in the first 5 min after glucose administration. For the measurement of the blood insulin concentration, blood samples (100 μL) were taken from the buccal veins at GTT0 and GTT5, and the plasma was stored at −80°C after centrifugation until it was used for insulin analysis.

#### Insulin tolerance tests

Intraperitoneal ITTs were performed as described previously [22] (Fig. 1-C). Briefly, the mice were fasted overnight (16 h) with free access to water. The mice were injected intraperitoneally with 0.075 U of recombinant human insulin (Humulin-R; Eli Lilly, Indianapolis, IN) per kilogram of body weight. Venous blood was obtained from the tail vein at 0, 5, 15, 30, 60, and 90 min after the injection and measured for glucose using an automatic glucometer (OneTouch Verio™ IQ meter or Medisafe FIT). The time points at 0 and 15 min post-injection were designated “ITT0” and “ITT15”, respectively.

#### Saline tolerance tests

Intraperitoneal saline tolerance tests (STTs) were conducted to assess whether the injection maneuver itself influenced blood glucose levels (Fig. 1-C). The mice were fasted overnight (16 h) with free access to water and then injected intraperitoneally with 75 mL of saline per kilogram body weight. Whole venous blood was obtained from the tail vein at 0, 5, 15, 30, 60, and 90 min after the injection and measured for glucose using an automatic glucometer (OneTouch Verio™ IQ meter or Medisafe FIT).

### Effects of interventions on the social hierarchy

We evaluated the influence of two types of interventions on the social hierarchy established among four co-caged mice and on the responses in the GTT and ITT: 1) dietary modification; and 2) chemogenetic suppression of BLA neuronal activity in the lowest-ranked mice in each cage. The methods for each intervention are described separately below (Figs. 1-B1 and 1-B2).

#### Intervention 1 – diet change

##### Diet

After being transported to Jikei University Laboratory Animal Facility, the mice were co-housed (four mice/cage) and fed “regular chow” provided by the animal supplier (3.40 kcal/g; CLEA Japan, Inc., Tokyo, Japan). From the fourth day of co-housing (15 +3 +2 +13 days before the diet change in Fig. 1-B1, i.e., Day −33), they were fed with “control chow” (D1250J; Research Diets, New Brunswick, NJ) composed of (in g% (kcal%)) 19 (20) protein, 67 (70) carbohydrate, and 4 (10) fat, giving a total of 3.8 kcal/g (Table 1-(1)). This was replaced with a “high-fat diet” (D12492J; Research Diets) composed of (in g% (kcal%)) 26 (20) protein, 26 (20) carbohydrate, and 35 (60) fat, giving a total of 5.21 kcal/g (Table 1-(2)) on Day 0 (Fig. 1-B2). The exact composition of the chow is listed in Table 1. The weights of the mice were measured almost daily during the habituation period and after the tube tests.

##### Pancreatic histology

**Fig. 3:**
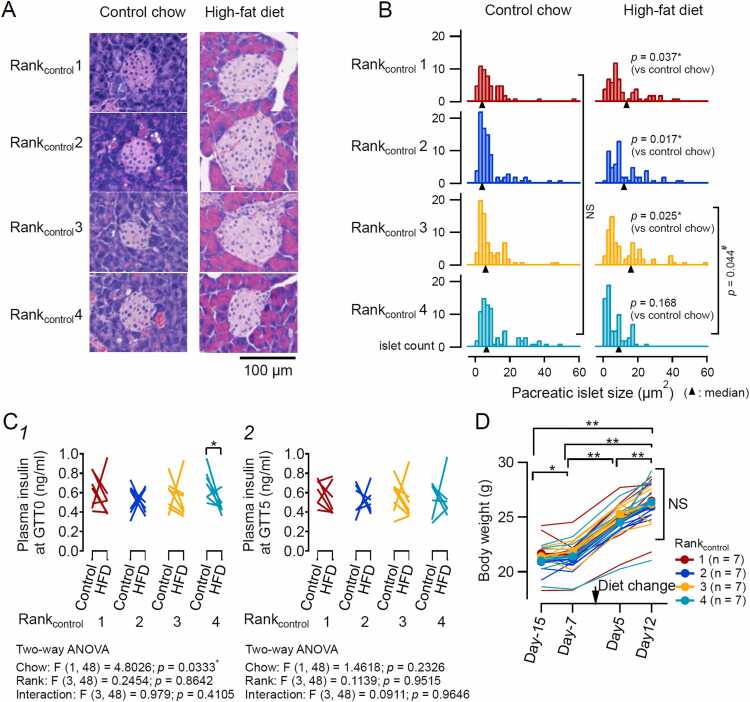
Relationships between the hierarchical rank and pancreatic islet size from control chow- and high-fat diet-fed mice. **A.** Representative H&E staining of the pancreatic islets of different rank_control_ mice fed control chow (left, Day −4) and a high-fat diet (right, Day 21). **B.** Distribution of pancreatic islet size in rank_control_1–4 mice before and after diet change. For control chow, rank_control_1, 2, 3, and 4 included 4, 5, 4, and 5 mice (56, 85, 72, and 77 islets), respectively. Under a high-fat diet, rank_control_1, 2, 3, and 4 included 7 mice (59, 58, 71, and 71 islets), respectively. Distributions significantly differed between pre- and post-diet change in rank_control_1 (*p* = 0.037*), rank_control_2 (*p* = 0.017*), and rank_control_3 (*p* = 0.025*), but not in rank_control_4 (*p* = 0.168; Kolmogorov–Smirnov test between pre- and post-intervention within each rank and between each rank_control_ group). Among ranks under a high-fat diet, rank_control_3 mice showed significantly larger islets compared with rank_control_4 mice (*p* = 0.044^#^). **C.** Basal plasma insulin concentration in individual mice. *p < 0.05. Paired *t*-test between the control chow and high-fat diet conditions. Results of two-way ANOVA in the basal plasma insulin concentration (GTT0) were as follows: two-way ANOVA, Chow: F (1, 48) = 4.8026; *p* = 0.0333*; Rank: F (3, 48) = 0.2454; *p* = 0.8642; Interaction: F (3, 48) = 0.979; *p* = 0.4105 (C1). Plasma insulin concentration at GTT5 in individual mice. Results of two-way ANOVA in the plasma insulin concentration at GTT5 were as follows: two-way ANOVA, Chow: F (1, 48) = 1.4618; *p* = 0.2326; Rank: F (3, 48) = 0.1139; *p* = 0.9515; Interaction: F (3, 48) = 0.0911; *p* = 0.9646 (C2). **D.** Rank_control_-wise plot of the body weight of mice with different ranks on Days −15, −7, + 5, and + 12. Mean (colored filled circles and thick lines) and individual body weight values (colored connected lines). The body weight significantly increased in the course of repeated measurements (Friedman test, *p < 0.05, **p < 0.005; n = 7 mice for the four rank groups) without any significant differences between different rank_control_ mice at each time point (Bonferroni post hoc correction; NS, *p* > 0.05).

In the dietary intervention experiments, after the GTT and ITT were performed under control chow and high-fat diet conditions, four mice in each rank order were selected and sacrificed for measurement of the pancreatic size. The pancreas was removed from each mouse after cervical dislocation on (1) Day −4, immediately after the ITT for the control chow-fed mice (18 mice from 5 cages; 2 mice accidentally died immediately after the GTT; Fig. 2-A), and (2) Day 21 after the last tube test for the mice being fed with high-fat diet (28 mice from 7 cages). The removed pancreases were submerged overnight in 10 % phosphate-buffered formalin and embedded in paraffin. Afterward, 2.5-µm-thick transverse sections were obtained, deparaffinized, hydrated, stained with hematoxylin and eosin (H&E), and covered with glass coverslips. The sections were imaged using a fluorescence microscope (BX63; Olympus, Tokyo, Japan). The areas of 8–18 islets in stained pancreatic slides (1 slide/mouse) were analyzed using ImageJ ver. 1.48 (National Institutes of Health, Bethesda, MD) in a blinded fashion [28] to construct the area distribution histogram (Fig. 3-B).

#### Intervention 2 – chemogenetic suppression of BLA neuronal activity

##### Viral vector injection

All of the mice used for the chemogenetic experiments received AAV solution injections into the right BLA prior to the assessment of hierarchical rank according to a previously described method [29]. The head of each mouse was fixed in a stereotaxic frame (Narishige, Tokyo, Japan), and the mouse was anesthetized with a mixture [30] of medetomidine hydrochloride (0.3 mg/kg) (Zenoaq; Orion Corporation, Espoo, Finland), midazolam (4.0 mg/kg) (Astellas, Tokyo, Japan), and butorphanol tartrate (5.0 mg/kg) (Meiji Seika Pharma, Tokyo, Japan). A midline longitudinal skin incision was made to expose the bregma and lambda sutures of the skull, and the peri-cranial connective tissues were removed.

##### Effect of hM4Di activation on rank and glucose metabolism

On Day 0, only the rank_pre-CNO_4 mouse received clozapine-N-oxide (CNO) and was returned to the same cage (Fig. 1-B2). Mice classified as rank 1, rank 2, or rank 3 received neither CNO nor vehicle (Fig. 5-B). Chemogenetic activation of hM4Di was induced by the intraperitoneal administration of CNO (3 mg/kg), which was dissolved in normal saline immediately before administration. The tube test was performed 30 min before and 30, 60, 90, 120, 180, and 240 min after each CNO administration, considering the time course of the effects of CNO in rodents [31], [32], [33] (Fig. 5-B2). Due to the potential off-target effects of CNO, either directly or after being metabolized to clozapine [34], vehicle comparisons were not performed in this study. Instead, comparisons of hM4Di activation effects were made between mice expressing hM4Di and those expressing EGFP [35].

A 500-nL viral solution containing 3 × 10^12^ genomic copies/mL of AAV5-CaMKIIa-hM4Di-mCherry (Addgene plasmid #50477) or 3 × 10^12^ genomic copies/mL of AAV5-CaMKIIa-EGFP (Addgene plasmid #50469) was randomly selected and injected into the right BLA (1.80 mm anterior, 3.4 mm lateral, and 4.8 mm ventral from bregma) [36], using a 30-gauge needle attached to a 2-μL Hamilton syringe controlled by a microinjection pump (UMP-4; World Precision Instruments, Sarasota, FL). The solutions also contained FluoSpheres (1.25 %, F-8794 or F-8795, 0.04 μm; Molecular Probes, Thermo Fisher Scientific, Waltham, MA) for post-experimental verification of the injection sites. In the mouse cohort with intra-BLA viral solution injection, the tube test began 20–27 days after the AAV solution injection (days −37 to −30 relative to the day of CNO injection; Fig. 1-B2). On the fifth day of the daily rank evaluation, each animal’s control rank (referred to as “rank_pre-CNO_”) was determined (Fig. 5-B and Figure S4). CNO was injected only into the mice with rank_pre-CNO_4 (the lowest-ranked mice in each cage) on Day 0. The tube test was restarted on Day 3 (Fig. 5-B), and the final tube test was performed 8 days later (Day 8). GTTs were performed on Days −6 and + 8, and ITTs were performed on Days −3 and + 12 (Fig. 5-B1).

##### Electrophysiological validation of the activation of chemogenetic receptors

After the experiments of intervention 2, we directly confirmed that CNO suppresses the excitability of BLA neurons in brain slices prepared from mice that underwent behavioral analysis using patch-clamp recording of membrane potential using the methods that we have previously published [29]. Briefly, we prepared acute 300-µm-thick brain slices containing the right BLA. Membrane potentials were recorded from mCherry- or EGFP-expressing neurons, and the resting membrane potential was measured. We applied CNO (5 µM) to the slice by adding CNO to the perfusion solution and observed its effect. The detailed methods for slice preparation, neuron visualization, and membrane current-recording using patch-clamp systems are described in the Supplementary Information.

##### Visual confirmation of fluorescent protein expression

After the final behavioral experiment, the mice were administered sodium pentobarbital (100 mg/kg, intraperitoneal; Somnopentyl, Kyoritsuseiyaku Corporation, Tokyo, Japan) and immediately perfused intracardially with ∼20 mL of ice-cold phosphate-buffered saline (PBS), followed by ∼50 mL of 4 % paraformaldehyde in 0.1 M phosphate buffer. The brains were removed and immersed in 20 % (wt/vol) sucrose in PBS for 1–2 days at 4°C. They were then sectioned into 50-μm or 200-μm coronal slices using a cryostat (CM1850; Leica Biosystems, Tokyo, Japan) or vibrating blade slicer (Pro 7; Dosaka Instruments, Kyoto, Japan), respectively. The sections were mounted on glass slides (Matsunami Glass Ind., Ltd., Osaka, Japan) and coverslipped.

The fluorescence of mCherry, EGFP, and FluoSpheres in the amygdala area was visualized using a fluorescence microscope (BX-63; Olympus) and visually confirmed after registering the slices to a stereotaxic atlas [36], using PowerPoint's image overlay function to align the images. The fluorescence signal boundaries were drawn on the atlas of the corresponding brain levels.

### Statistics

Statistical analyses were performed using EZR, a modified version of R Commander designed to add statistical functions frequently used in biostatistics [37]. The following approaches were used to test the null hypotheses for each independent comparison:

#### Comparison of means and variance

Comparisons between different rank groups were made by one-way analysis of variance (ANOVA), followed by Tukey’s multiple comparisons of means. A paired *t*-test was employed to analyze plasma insulin concentrations and glucose concentrations at different time points. The Wilcoxon signed-rank test was performed to compare changes in ranks across different time points. Two-way ANOVA was performed to determine statistical differences in blood glucose levels across pre-CNO ranks and time points in glucose and insulin tolerance tests. In addition, two-way ANOVA was performed to determine statistical differences in blood insulin levels across rank_control_s and time points in glucose tolerance tests. Body weight was analyzed using non-parametric statistical tests because the data did not follow a normal distribution. Difference in distribution was analyzed with Kolmogorov–Smirnov test. To assess differences between rank groups at each time point (Days –15, –7, 5, and 12), the Kruskal–Wallis test was performed. To evaluate longitudinal changes in body weight across time within the same rank group, the Friedman test was applied, followed by Bonferroni post-hoc correction. A probability < 0.05 was considered significant.

#### Hierarchical cluster analysis

**Fig. 4:**
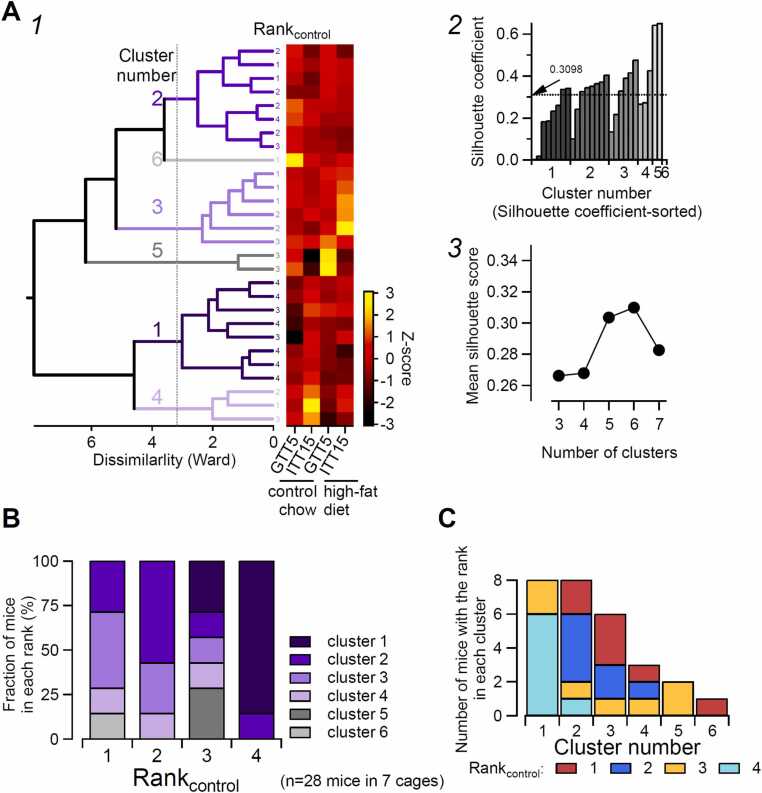
Hierarchical cluster analysis of mice using glucose tolerance and insulin tolerance responses. **A.** Hierarchical clustering of GTT and ITT data. Blood glucose concentrations (at GTT5 and ITT15) during feedings with control chow and high-fat diet (28 mice from 7 cages; Y-axis) were used to calculate dissimilarity distances (X-axis in the left plot). The dendrogram (A1, left) was constructed using Ward’s method. Silhouette scores were calculated for different cluster numbers (A2), and a six-cluster classification yielded the highest score (A3). The rank_control_ of each mouse is shown to the right of the dendrogram. The heatmap (right of A1) indicates the Z-scores of the four variables (X-axis) from the 28 mice. The broken line in A2 is the mean silhouette score assuming 6 clusters. **B.** Distribution of mice across clusters within each rank. Among the 28 mice from 7 cages, 86 % of rank_control_4 mice were classified into cluster 1, whereas no cluster 1 mice were present in rank_control_1 or 2. **C.** Biased relationship between cluster number and social rank. Most of cluster 1 consisted of rank_control_4 mice, and no rank_control_4 mice were classified into clusters 3–6 (see text for details).

Hierarchical cluster analysis of blood glucose concentrations using the values obtained in GTTs and ITTs in each mouse from 7 cages (28 mice) was used to examine whether these values could predict the rank of the mice (Fig. 4). Blood glucose values obtained at GTT5 and ITT15 for the control chow and high-fat diets were used to calculate Euclidean distances (Ward’s method) after converting to Z-scores and used for drawing a dendrogram using the code implemented in Igor 9. The optimum number of clusters was examined using silhouette score estimation [38].

#### Schema drawing

**Fig. 5:**
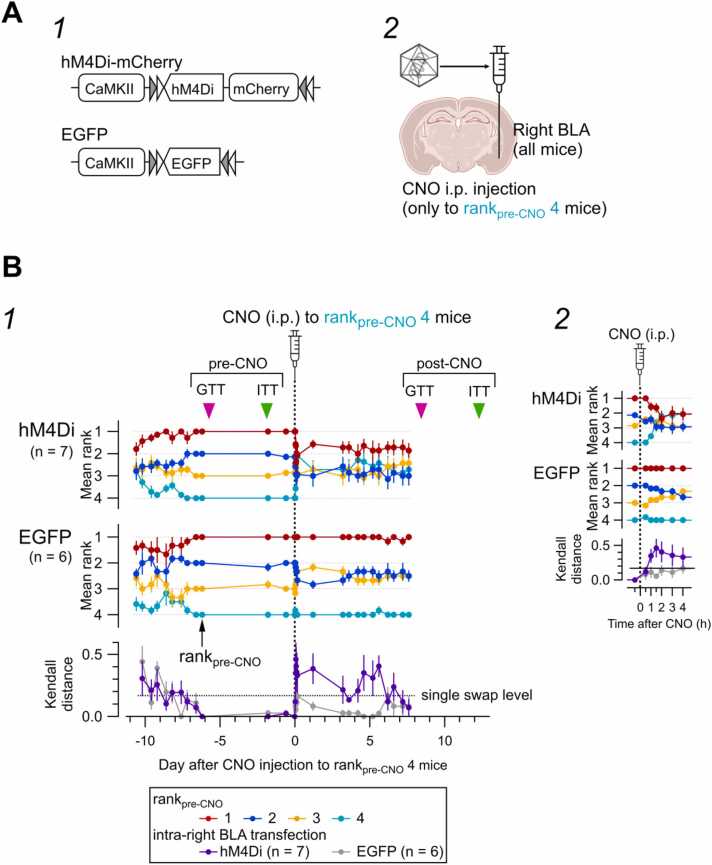
Effect of chemogenetic inhibition of neurons in the basolateral amygdala and surrounding area on the hierarchical rank. **A.** Schema of the AAV injection protocol for expressing hM4Di receptors and EGFP in the principal neurons of the right basolateral amygdala (BLA). A1, ITR-ITR sequence of the AAV for the Cre-dependent expression of hM4Di-mCherry (DREADD) or EGFP (control to DREADD) in excitatory neurons in the BLA. A2, Schema of AAV injection into the right BLA. **B.** The top two graphs show the time course of the mean rank for each rank_pre-CNO_ at each assessment for hM4Di (top plots, 7 mice from 7 cages) and EGFP (middle plots, 6 mice from 6 cages). Circle and line colors represent the rank_pre-CNO_. The mean Kendall distance of the ranks between each consecutive tube test in each mouse (bottom plots of B1 and B2). The horizontal broken line indicates the Kendall distance with a single swap (see Fig. S1-A) in hM4Di (light purple, n = 28) and EGFP (light gray, n = 24) groups. Mean ± SEM. The horizontal axis indicates the day relative to CNO injection common for these three graphs. Tube tests were performed daily from Days −11 to −7 and then from Days −2 to + 8. The stable ranks of individual mice on Day −7 were designated “rank_pre-CNO_”. CNO was administered only to rank_pre-CNO_4 mice on Day 0 (dotted vertical line). The timings of the glucose test (GTT_pre-CNO_, Day −6, magenta arrowhead; and GTT_post-CNO_, Day 8, magenta arrowhead) and insulin test (ITT_pre-CNO_, Day −2, green arrowhead; and ITT_post-CNO_, Day 12, green arrowhead) are indicated with arrowheads on the top. B2. Time-extended versions of the time course of the mean rank of each mouse for the period 0.5 h before CNO injection to 4 h post-injection, during which time the tube test was repeated every 0.5 h or 1 h.

The coronal brain schemas are adapted from “Mouse” by BioRender.com (2023) (for Fig. 5).

## Results

### Influence of diet on the established social rank and glucose metabolism

#### Effect of a changed diet on social rank

We evaluated the rank order of the four mice co-housed in a cage using the tube test. We determined the tube-test rank of each mouse as a proxy for the hierarchical order formed among the co-housed mice based on the “win-lose” results of six games (called a “session”) between pairs of mice from a cage (Figure S1-A). Figure S2 shows the time course of the color-coded rank of all mice in the seven cages across sessions (Y-axis) (ranks 1–4, four colors) from Day −13 (X-axis) to Day 21, while Fig. 2-A shows the time course of the cage-wise mean and standard error of each mouse's rank. The “rank_control_” (rank_control_1, 2, 3, and 4) within each cage was defined as an attribute of each mouse according to the rank on Day −8 on the abscissa of Fig. 2-A (dashed line in Figure S2) as the rank of each mouse was stabilized within ∼5 days of a repeated tube test (Fig. 2-A, Figure S2).

Replacing the control chow with the high-fat diet on Day 0 perturbed the ranks of the mice (the timing of the diet change is shown as a vertical dashed line and a change in the background color of the graph from white to pink in Fig. 2-A and Figure S2). The shift in rank started within 2 days post-diet change, after which unstable state continued. Figure S2 indicates that the diet change strongly destabilized and perturbed the rank hierarchy of the mice. Upon changes to the high-fat diet, the rank of the rank_control_1 mice significantly lowered (Fig. 2-A, red lines and markers; Wilcoxon signed-rank test, V = 21, *p* = 0.0034; a comparison between days −9 and 12), whereas the ranks of rank_control_2, 3, and 4 mice did not significantly change (Wilcoxon signed-rank test, V = 7, 0, and 0, *p* = 0.484, 0.053, and 1, respectively). To evaluate how the ranks of each mouse were modified in each daily measurement, we calculated the KD between each daily measurement (Fig. 2-A, bottom). The KD increased after the diet change, indicating that the individual rank became variable (Fig. 2-A, bottom). Notably, the rank of the rank_control_4 mice (i.e., the lowest-ranked mice under the control chow condition) was less perturbed than that of the rank_control_1, 2, and 3 mice (green plot in Figure S2).

#### Diet changes affect the glucose- and insulin-dependent regulation of blood glucose in a social rank-associated manner

As shown with colored arrowheads in Fig. 2-A and Figure S2, we performed GTTs, ITTs, and STTs to evaluate the homeostatic responses to exogenous glucose, the direct influence of exogenous insulin, and the effect of the saline injection maneuver, respectively, on the plasma glucose level in each mouse with a determined rank order and observed the effect of the diet change on Day 0.

The time courses of the blood glucose level responses differed drastically between the control chow (Fig. 2-B1) and high-fat diet (Fig. 2-B3) conditions, as demonstrated in previous studies [24], [39], [40], [41], [42]. In particular, these differences between the diet conditions were manifested in the early phase (0–15 min) after the glucose injection (Fig. 2-B1 and B2), suggesting that the diet change affected the rapid homeostatic regulation of blood glucose.

In addition, these chow-dependent glucose responses were associated with the animal’s rank. The blood glucose level at 5 min after the glucose injection (GTT5) significantly differed between mice of different ranks under the control chow condition (one-way ANOVA, F(3, 42) = 4.903, *p* = 0.005, Fig. 2-B2), with a significantly lower glucose level in rank 4 mice than in rank_control_1 and 3 mice (Tukey multiple comparisons of means for rank_control_1 vs 4, *p* = 0.036*; rank_control_ 3 vs 4, *p* = 0.004**). These rank-related differences were not evident under the high-fat diet condition (Fig. 2-B4). The fasting blood glucose levels (i.e., GTT0 and ITT0) did not differ significantly between mouse groups at different ranks, regardless of the diet condition (GTT0 at control chow: one-way ANOVA, F (3, 44) = 1.054, *p* = 0.378, Fig. 2-B2; ITT0 at control chow: one-way ANOVA, F (3, 42) = 0.012, *p* = 0.998, Fig. 2-C1; GTT0 at high-fat diet: one-way ANOVA, F (3, 24) = 0.857, *p* = 0.477, Fig. 2-B4; ITT0 at high-fat diet: one-way ANOVA, F (3, 24) = 0.197, *p* = 0.897, Fig. 2-C2). In addition, these increases in glucose levels were not likely to be due to sympathetic and adrenal activation caused by the injection maneuver (holding and needle insertion), as an equal-volume intraperitoneal injection of saline (STT) failed to cause rank-associated blood glucose responses (Figure S3). We also analyzed the change in glucose levels caused by exogenous insulin (ITT). Fig. 2-C shows the rank-associated differences in the ITT parameters. The blood glucose levels of rank_control_4 mice at 15 min post-insulin injection (ITT15) were significantly lower than those of rank_control_1 and rank_control_2 mice (one-way ANOVA, F = 5.104, *p* = 0.004; Tukey’s multiple comparisons of means for rank_control_1 vs 4, *p* = 0.005; rank_control_2 vs 4, *p* = 0.021) (Fig. 2-C1; ITT15 for control chow).

In summary, these GTT and ITT results suggest that, in the lowest-rank mice under the control chow condition, an increase in blood glucose is rapidly returned to the basal level, and insulin is more effective at rapidly decreasing the blood glucose level, whereas such rank-related particularities in rapid glucose metabolism are obscured in the mice fed a high-fat diet (Fig. 2-C).

#### Pancreatic size and fasting plasma insulin level were associated with the diet in the lowest-rank mice

Dietary fat content influences the morphological features of the pancreas and insulin release capability [43]. We analyzed the size of the pancreatic islets in relation to each mouse’s diet and rank. Figs. 3-A and 3-B show images of the sampled pancreas from the mice of each rank_control_ and the distribution of the size of islets identified in representative sections, respectively.

The distribution of pancreatic islet size did not differ between those sampled from the mice under the control chow and high-fat diet, and among those from different rank_control_ mice in the control chow (Fig. 3-B, left), suggesting that the basal islet size has a limited contribution to the rank hierarchy. However, the islet size distribution differed significantly between control chow and high-fat diet for rank_control_1–3 mice, which was not the case for rank_control_4 mice. In the mice with high-fat diet, the islet size distribution of rank_control_4 mice significantly differed from that of rank_control_3 mice (Fig. 3-B, right), whereas we did not find any significant difference between other combinations for both the control chow and high-fat diet.

We then asked whether baseline plasma insulin levels differed among mice of different ranks and whether these levels were modified by the diet change (Fig. 3-C). First, we compared baseline insulin concentrations at GTT0 between mice of different ranks under control chow (Control) and a high-fat diet (HFD; Fig. 3-C1). The diet change did not significantly alter plasma insulin levels in mice belonging to rank_control_1, 2, or 3, but it significantly decreased plasma insulin levels in rank_control_4 mice only (Fig. 3-C1). Such diet-induced changes in plasma insulin concentration were not observed at GTT5 (Fig. 3-C2). These results support the notion that mice belonging to rank_control_4 exhibit specific characteristics in the regulation of blood glucose through insulin secretion, which are affected by diet.

The body weights of the different rank_control_ mice did not differ significantly at any stage, regardless of the type of the diet (repeated-measures ANOVA, NS, *p* > 0.05; Fig. 3-D), despite significant increases in body weight over the course of repeated measurements, as expected (Fig. 3-D; Friedman test, n = 7 mice for each of the four ranks).

#### Rapid glucose regulation with different diet types is associated with the rank_control_ of the mouse

The above results regarding the rapid GTT and ITT responses in distinct diets was associated with the rank_control_ of each mouse. This result was unexpected, as glucose metabolism and social rank are, in principle, separate attributes of each mouse. As shown above, diet-induced changes in glucose metabolism parameters varied among mice, and shifts in social rank also differed across individuals. We therefore asked whether parameters reflecting glucose responses in the GTT and ITT under different dietary conditions were associated with a mouse’s social rank under the control diet, which would reflect an individual behavioral trait. For this purpose, we applied hierarchical cluster analysis to group the mice into different clusters using only the glucose metabolism-related parameters and asked whether a specific cluster represents the specific rank of the mice (Fig. 4). The dissimilarities of the clusters were calculated according to Ward’s method and then used to create the dendrogram shown on the left in Fig. 4-A1. To the right of the dendrogram is the heat map display of blood glucose level at GTT5 and ITT15 for the control chow and high-fat diet mice (abscissa). The dendrogram and heatmap share the same ordinates corresponding to the 28 mice used in this experiment of intervention 1. Using these glucose metabolism parameters, the mice could be classified into several clusters as seen on the dendrogram (Fig. 4-A1). Apparent observation of the dendrogram gives impression that they could be classified roughly into 3–6 clusters. To estimate the best dissimilarity threshold for clustering, we calculated the Silhouette coefficient, which provides estimates as to how well the groups are separated [38] (Fig. 4-A2 and A3). Fig. 4-A2 is an example result of a Shilhouette analysis showing that the classification into 6 groups, as shown with the vertical broken line shown in Fig. 4-A1 gives the mean Shilhouette coefficient of 0.3098, which is the largest of the results with classifications into 3–7 groups (Fig. 4-A3). Based on this result, we assumed that the mice could be grouped into 6 clusters.

Fig. 4-B shows the fraction of the mice belonging to each cluster in each rank_control_ based on the real data of the intervention 1 cohort. Notably, 86 % of the rank_control_4 mice (24 of 28 mice in 6 of 7 cages) were classified as belonging to cluster 1 (Fig. 4-B, rank_control_4). This fraction was far larger than the estimated fraction of 1.1667 of 7 mice (16.667 %), assuming that 28 mice were randomly classified into 7 sets of 4 rank groups and each mouse were assigned to any of 6 clusters. This result implies that the clustering of the mice using only the rapid glucose responses can result in biased classification, where the most of the lowest-rank mice (rank_control_4) belong to the same cluster (cluster 1). On the other hand (Fig. 4-C), 75 % (6 of 8) of the mice classified on the basis of glucose metabolism as cluster 1 were rank_control_4 mice, and all mice belonging to cluster 5 (n = 2) and cluster 6 (n = 1) were rank_control_3 and rank_control_1 mice, respectively. These results suggest that there is a potential association among glucose metabolism, diet, and the social rank of individual mice.

### Influence of reduced amygdala neuronal excitability on glucose metabolism and social rank

#### Effect of hM4Di receptor activation in the BLA and surrounding areas on social rank and glucose metabolism

The above results indicated that manipulation of the nutritional conditions that affect glucose metabolism also markedly affects the rank of the cage mate. Next, we explored the reverse question: does a neuronal intervention known to affect hierarchical rank order influence glucose metabolism?

For this purpose, we used chemogenetic techniques to artificially modulate neuronal activity by injecting an artificial ligand into mice transfected with artificial receptors to modify the hierarchical structures [29], [44], [45], [46]. We targeted the right BLA, a region implicated in subordinate behavior in response to dominant conspecifics that receives excitatory inputs from layer II–III neurons of the dmPFC, a core structure regulating social rank-related behaviors [21]. We hypothesized that suppressing the excitability of principal neurons in the BLA would attenuate subordinate behaviors in lower-ranking mice and consequently increase their relative social rank [21].

As explained in the Methods section (Fig. 1-B2), all four mice in each of the seven cages received an AAV vector for the CaMKII-dependent expression of hM4Di, whereas those in the other six cages received an AAV vector for the expression of EGFP in the BLA from −38 to −31 days (= 0–7 days + 15 + 3 + 2 + 11 days) before CNO injection (Fig. 1-B and Fig. 5-A). The rank of individual mice, as evaluated on Day −6, on which the rank of the mice in each cage was largely stabilized (Figure S2), was used to represent the control rank of the mouse and described as rank_pre-CNO_. A hM4Di ligand, CNO, was administered only to the rank_pre-CNO_4 mice (Figs. 5-A2 and 5-B) according to the result of the last tube test before the CNO injection. We used this approach because, at the moment of transfection, which was > 20 days before the beginning of the tube test (Fig. 1-B2), the rank of each mouse had not yet been identified.

In these mice with hM4Di expression (hM4Di group), CNO injection only to the rank_pre-CNO_4 mice perturbed the entire hierarchical structure formed among the four cage mates (top panels in Fig. 5-B and S4), accompanied by an abrupt increase in the KD (bottom of Fig. 5-B), indicating that CNO injection destabilized the rank of the mice expressing hM4Di in the BLA and surrounding areas. These changes occurred immediately, as early as 1 h postinjection (Fig. 5-B2). These changes did not occur in the EGFP group (middle panels in Figs. 5-B1 and 5-B2 and bottom panel in Figure S4).

**Fig. 6:**
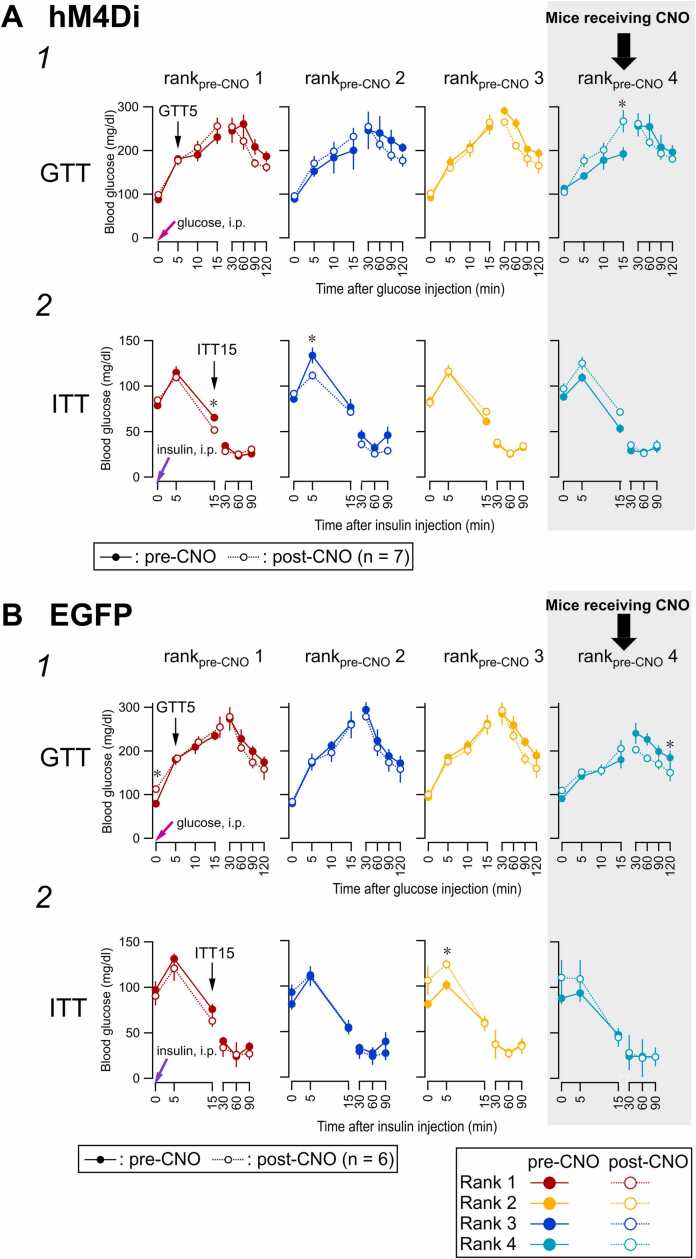
Rank_pre-CNO_-wise display of time course of blood glucose levels in the GTT and ITT before and after inhibition of the amygdala neurons in rank_pre-CNO_4 mice. Glucose metabolism in the (A) hM4Di (n = 28 mice from 7 cages) and (B) EGFP control (n = 24 from 6 cages) groups. Glucose (A1 and B1) and insulin (A2 and B2) tolerance tests in hM4Di (A1 and A2) and EGFP (B1 and B2) mice. GTT in A1 and B1, time course of blood glucose concentrations immediately before (GTT0) and 5–120 min after intraperitoneal glucose injection at pre-CNO (Day −6, filled circles) and post-CNO (Day 8, open circles). ITT in A2 and B2, time course of blood glucose immediately before (ITT0) and 5–90 min after intraperitoneal insulin injection at pre- and post-CNO. *, *p* < 0.05 (paired *t*-test). GTT and ITT were performed in the same animals for each of hM4Di and EGFP control groups (see Fig. 5-B1).

**Fig. 7:**
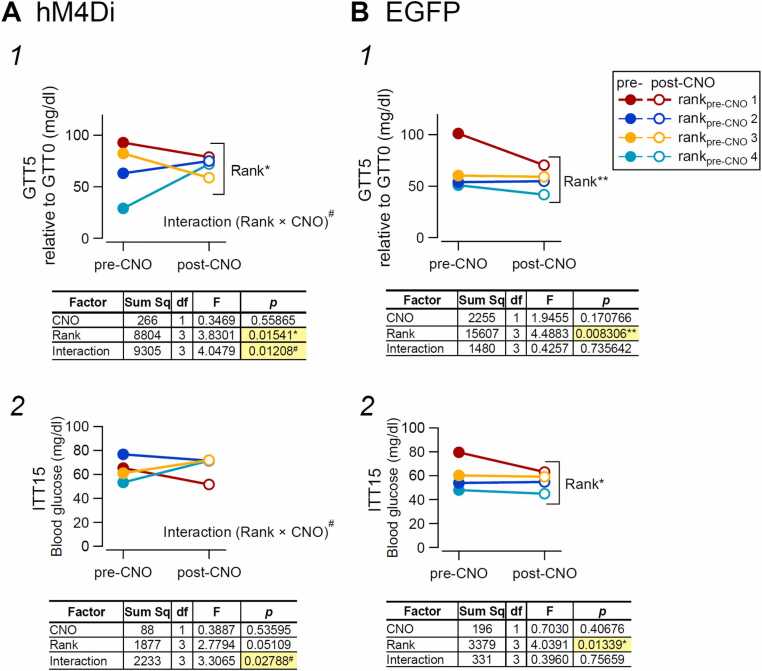
Effect of chemogenetic inhibition of neurons in the BLA and surrounding areas on the relationship between social rank and glucose metabolism. Glucose metabolism in the hM4Di (n = 28 mice from 7 cages, (A)) and EGFP control (n = 24 from 6 cages, (B)) groups. Glucose (A1 and B1) and insulin (A2 and B2) tolerance tests in hM4Di (A1 and A2) and EGFP (B1 and B2) mice. GTT and ITT were performed in the same animals (see Fig. 5, Fig. 6). **A**. Two-way ANOVA was used to assess the effects of rank_pre-CNO_ and CNO treatment on changes in Δglucose (at GTT0 relative to that at GTT5; A1) and ITT15 (A2) in individual hM4Di-expressing mice. For Δglucose (A1), there was a significant main effect of rank (F (3, 48) = 3.8301, *p* = 0.01541*) and a significant rank × CNO interaction (F (3, 48) = 4.0479, *p* = 0.01208^#^), whereas the main effect of CNO was not significant (F (1, 48) = 0.3469, *p* = 0.55865). For ITT15 (A2), the main effect of rank did not reach significance (F (3, 48) = 2.7794, *p* = 0.05109) and there was no main effect of CNO (F (1, 48) = 0.3887, *p* = 0.53595); however, the rank × CNO interaction was significant (F (3, 48) = 3.3065, *p* = 0.02788^#^). Sum Sq, sum of squares. **B.** Two-way ANOVA was used to assess the effects of rank_pre-CNO_ and CNO treatment on changes in Δglucose (values at GTT5 relative to that at GTT0; B1) and ITT15 (B2) in individual EGFP-expressing mice. For Δglucose (B1), there was a significant main effect of rank (F (3, 40) = 4.4883, *p* = 0.008306**), whereas the main effect of CNO was not significant (F (1, 40) = 1.9455, *p* = 0.170766), and no rank × CNO interaction was observed (F (3, 40) = 0.4257, *p* = 0.735642). For ITT15 (B2), the main effect of rank was significant (F (3, 40) = 4.0391, *p* = 0.01339*), while the main effect of CNO was not significant (F (1, 40) = 0.7030, *p* = 0.40676), and the rank × CNO interaction was not significant (F (3, 40) = 0.3960, *p* = 0.75659).

We then compared the GTT and ITT responses before and after CNO injection and among different social ranks (Fig. 6, Fig. 7).

Fig. 6 shows the time courses of blood glucose concentrations during the GTT (Fig. 6-A1, B1) and ITT (Fig. 6-A2, B2) in mouse groups classified as ranks 1–4 based on pre-CNO tube test results. Among these cohorts, only mice classified as rank_pre-CNO_4 received a single CNO injection, according to the tube test classification (thick arrows in Fig. 6). In rank_pre-CNO_4 mice, the blood glucose level at GTT15 was significantly increased following CNO injection. This finding indicates that suppression of amygdala and surrounding area activity alters glucose metabolism, at least in rank_pre-CNO_4 mice, representing a direct effect of inhibiting amygdala activity in these individuals.

In contrast, in mice classified as rank_pre-CNO_1, 2, and 3, differences between pre- and post-CNO conditions suggest that changes in hierarchical rank resulted in altered glucose responses. Specifically, ITT15 after CNO injection was significantly reduced in rank_pre-CNO_1 mice, and ITT5 after CNO injection was significantly reduced in rank_pre-CNO_2 mice. These results indicate that even without direct modulation of basolateral amygdala (BLA) and surrounding area activity, alterations in hierarchical structure—as shown in Fig. 5-B and Figure S4—can modulate glucose metabolism.

We next asked whether changes in glucose levels at GTT5 and ITT15 were affected by activation of hM4Di receptors by CNO injected into rank_pre-CNO_4 mice. To address this, we performed a two-way ANOVA to examine whether there was a significant interaction between the effects of rank and CNO (Fig. 7).

In the hM4Di group, in which CNO was injected only into rank_pre-CNO_4 mice, two-way ANOVA of the GTT responses revealed a strong and significant interaction between the effects of CNO and post-glucose-load changes in blood glucose levels (Fig. 7-A1). In addition, there was a significant difference in glucose levels relative to GTT0 among mice of different ranks that was associated with the presence of CNO, despite the absence of a significant main effect of CNO alone (Fig. 7-A1).

For ITT responses in hM4Di-expressing mice, two-way ANOVA revealed a significant interaction between the effects of CNO and changes in plasma glucose levels at ITT15 (Fig. 7-A2), suggesting that the effect of CNO injected into rank_pre-CNO_4 mice on insulin-induced plasma glucose changes was associated with the rank_pre-CNO_ of individual mice. Although differences before and after CNO administration, as well as differences among ranks, did not reach statistical significance (p > 0.05; Fig. 7-A2), these results suggest that glucose responses—particularly at GTT5—were significantly associated with the rank of individual mice established before and after CNO injection into rank_pre-CNO_4 mice (Fig. 7-A1).

In contrast, in the EGFP control group (Fig. 7-B), although significant differences in GTT5 (Fig. 7-B1) and in ITT15 (Fig. 7-B2) responses were observed among mice of different ranks, CNO did not exert significant main effects on GTT5 and ITT15 responses, and there was no significant interaction between the effects of CNO and rank_pre-CNO_ (Fig. 7-B2). Thus, suppressing the neuronal activities in the BLA and surrounding areas drastically altered the hierarchical order formed among co-housed mice even at > 7 days after intervention, which was accompanied by significant changes in blood glucose levels at GTT5 and ITT15 with the condition that ranks are different. In contrast, in mice with EGFP expression in place of hM4Di, this interaction was not observed (i.e., the glucose responses differed between ranks, regardless of the effect of CNO).

After the experiments, we confirmed that neurons in the BLA and surrounding areas transfected with hM4Di, but not those transfected with EGFP, consistently showed hyperpolarization and attenuated excitability upon direct application of CNO (Figure S5-A–D) and that mCherry or EGFP was expressed throughout the BLA and the areas surrounding the BLA (Figure S5-E).

## Discussion

Using a recently established behavioral test (tube test) that can robustly determine the hierarchical rank of mice co-housed in the same cage [18], [19], [20], [47], [48], we evaluated the impact of two types of interventions on the individual rank and glucose metabolism of mice with identified social ranks: (1) a dietary modification, and (2) inhibition of neuronal activity in the BLA and surrounding areas. We showed the following: (1) an experimental change in the fat content of chow immediately modifies the hierarchical rank of each mouse (Fig. 2-A); (2) the individual trait of glucose metabolism is also modified by the diet change, as expected, which is related with the rank of the individual mice (Figs. 2-B and 2-C); (3) these rank-associated changes in glucose metabolism with chow replacement mostly consisted of changes in rapid glucose responses to glucose or insulin injection and were associated with pancreatic islet size, particularly in the lowest-rank mice (Figs. 2-B and 3); (4) the mice could be classified into clusters using only these rapid glucose responses, and this classification depended significantly on the rank of the mouse group (Fig. 4-A); and (5) the chemogenetic inhibition in the lowest-rank mice of BLA and surrounding area neurons, which have been shown to underlie subordinate behaviors [21], altered the hierarchy among co-housed mice and also significantly modified the glucose metabolism of mice at different ranks (Fig. 5, Fig. 6, Fig. 7, and S4). On the basis of these observations, we conclude that the established hierarchy among co-housed mice and individual traits of rapid glucose metabolism are mutually influenced by diet composition and amygdala activity. The underlying mechanisms and functional implications are discussed in the subsequent sections.

### Individual traits of glucose metabolism were associated with the rank of the individual mouse

This study is the first to demonstrate that dietary modification has an immediate and profound influence on the social hierarchy established among co-housed mice. Hierarchical reorganization began within a few days after the diet switch and stabilized within approximately 1 week. This change primarily reflected destabilization of the higher-ranking positions (e.g., rank_control_1–3), whereas the lowest-ranking mice (rank_control_4) remained remarkably stable (Fig. 2 and Figure S2).

Diet-induced alterations in glucose homeostasis are well documented [24], [39], [40], [41], [42], [43], [49]. Even short-term high-fat feeding (2–7 days) impairs early-phase glucose clearance by inducing insulin resistance in skeletal muscle and liver and by altering hepatic insulin signaling [42], [50], [51], thereby affecting the fast glucose response measured during the early time points of a GTT. Our findings on the changes in glucose metabolism are essentially in line with the results of these studies but further indicate that they strongly depend on the rank of the individual animal, which has never been examined.

In particular, under the control chow condition, rank 4 mice displayed the smallest increase in glucose at GTT5 (Figs. 2-B1 and 2-B2) and the largest decrease at ITT15 (Fig. 2-C1), despite having similar fasting glucose levels at GTT0 and similar body weights to rank 1–3 mice. In addition, high-fat feeding for 21 days in rank_control_4 mice did not significantly affect the median islet size, unlike rank_control_1, 2, and 3 mice that showed a significant increase (Figs. 3-A and 3-B). These observations are in line with an interpretation that the glucose-stabilizing capacity and the morphofunctional specificity of the islet and insulin-releasing mechanisms at various dietary conditions are under distinct regulatory mechanisms, likely involving distinct central regulation, in the mice at the lowest hierarchy compared to more dominant mice. However, glucose homeostasis is regulated by multiple processes, including hepatic glucose production (gluconeogenesis), peripheral glucose utilization or uptake, and insulin secretion. The ITT data, in particular, suggest that altered glucose uptake—potentially in an insulin-dependent manner—also contributes to the observed phenotype under distinct dietary conditions, as well as under conditions of altered central activity. Identifying the mechanisms underlying the links between the determinants of social hierarchy and glucose regulation over a longer time course will therefore be an important subject for future investigation.

Because basal glucose levels and body weight did not differ among ranks (GTT0 in Fig. 2, Fig. 3), these results suggest that hierarchical rank influences fast β-cell–driven responses rather than chronic systemic metabolic parameters, particularly under control chow conditions. This aligns with previous observations that social rank is not dependent on body weight [18], [48] and that body weight is not a determinant of hierarchical order in the tube test [52]. These observations are reminiscent of reports in primates and humans [53], [54], [55] showing that individual differences in glucose regulation may be linked to the social subordination of each individual. Collectively, these findings indicate a close association between social rank, individual traits of rapid glucose regulation and pancreatic responsiveness, and that dietary fat content modulates these traits in a rank-related manner.

### Dietary modification immediately alters the central regulation of hierarchy and glucose metabolism

The rapid reorganization within a few days to a week of social hierarchy following dietary modification is another novel finding of this study. This fact might suggest that nutrient-derived signals act on the activity of the brain network underlying the determination of dominant and subordinate behaviors before systemic metabolic changes develop.

Glucose metabolic parameters typically require several weeks to respond to dietary changes, indicating that the immediate rank shift is unlikely to have arisen from early insulin resistance or β-cell remodeling, as suggested by the changes found in islet size and glucose metabolism. The consumption of a high-fat diet for more than 4 weeks induces anxiolytic-like behaviors accompanied by increased levels of mBDNF in the mPFC, a brain region strongly associated with psychosocial traits and behavioral tendencies [13]. Although the time scale differed between that study and ours, it is possible that an upregulation of bioactive messengers in the brain in response to the diet change might underlie the altered social hierarchy. Remarkably, a single 24-h exposure to a high-fat diet upregulates inflammatory mediators and immune cells not only in the gut and nodose ganglion but also in the hypothalamus [15]. This finding is also in line with the potential involvement of inflammatory processes in the short-latency effects of a high-fat diet on the fast glucose homeostasis [42]. A recent study demonstrated that social hierarchy, as evaluated by the tube test, significantly affects vulnerability to stress-induced posttraumatic stress disorder (PTSD) through mechanisms involving microglial inflammation in the mPFC [56]. Future work should examine whether such inflammatory processes also underlie the diet-mediated changes in glucose homeostasis. In addition, the high-fat diet may have affected the neural network underlying the autonomic regulation of glucose homeostasis. For example, even a short-term dietary manipulation exerts marked effects on the network: consumption of a high-fat diet for only 3–5 days enhances excitatory synaptic transmission in the dorsal motor nucleus of the vagus nerve, a key site linking brain activity and parasympathetic outflow [14].

It is worth noting that nutrients can alter brain activity even within minutes. For example, intraduodenal infusion of fat, glucose, or amino acids rapidly suppresses the firing of agouti-related protein (AgRP) neurons in the arcuate nucleus, an effect attenuated by subdiaphragmatic vagotomy [57]. This result indicates that dietary signals are rapidly detected by vagal afferents and transmitted to the central autonomic network (CAN). High-fat feeding also elevates circulating corticosterone [58] and impairs gastric vagal afferent signaling [59], indicating that nutritional status modulates neuroendocrine pathways governing arousal, motivation, and stress coping. These fast-acting routes provide a plausible mechanism for the post-nutrient change in rank destabilization observed in higher-ranking mice: disruption of vagal or CAN activity may transiently impair behavioral performance during competitive interactions. In contrast, rank_control_4 mice—likely in a parasympathetic-dominant state—may be less affected, consistent with their stable hierarchical positions. This interpretation is further supported by the finding that chronic vagal afferent activation enhances fast glucose clearance without altering fasting glucose [7], reminiscent of the phenotype observed in rank_control_4 mice.

The CAN interacts closely with circuits mediating dominance and subordination. Recent studies have identified the dmPFC, BLA, dorsal raphe nucleus (DRN), and periaqueductal gray (PAG) as principal circuits determining social rank [21]. Projections from the dmPFC to the anterior BLA promote subordinate behaviors, whereas those from the dmPFC to the PAG and/or DRN promote dominance. The anterior BLA strongly regulates activity in the central amygdala (CeA) [60], whose medial subdivision projects to the PAG and DRN [61], [62]. This provides a pathway through which emotional threat prediction may modulate autonomic output to peripheral organs, including the pancreas. The amygdala circuit, which has strong bidirectional interactions with the prefrontal cortex, also regulates vagal parasympathetic outflow and controls glucose metabolism [63], [64], [65].

A plausible interpretation is that the better fast glucose regulation of rank 4 mice may facilitate behavioral strategies that prioritize danger avoidance and acceptance of subordinate status. In contrast, impaired fast glucose handling in higher-ranking mice under a high-fat diet may diminish their capacity to maintain dominance. A major future question is how dominance-related circuits (dmPFC–BLA–CeA–PAG–DRN) interact with the CAN to regulate β-cell responsiveness and glucose homeostasis. Understanding this link may illuminate how metabolic and social factors jointly shape individual behavioral phenotypes.

Interestingly, in both behavioral traits and glucose metabolism, rank-4 mice consistently exhibited distinct characteristics compared with mice at higher ranks. One possible interpretation of this specificity in the most subordinate mice is that they may have developed a specific survival strategy in the home cage. It is well known that chronic social defeat stress induces potent stress-related physiological and psychological responses. Thus, it might be also possible to argue that depression-like effects induced by chronic social defeat, rather than social hierarchy per se, may be the primary driver of the observed changes in glucose metabolism. For example, chronic social defeat induced by forced encounters with a physically dominant strain for 10 days has been reported to cause hyperglycemia accompanied by hypercortisolemia, adrenal hyperplasia, and hyperphagia, ultimately leading to impaired spatial memory [66].

However, this possibility is less likely as the tube test used in the present study and hierarchical order formed among co-housed mice would not be comparably stressful. We did not observe any mice exhibiting aberrant behavior or bodily injury in either the tube-test arena or the home cage. Indeed, Fan et al. reported, using the same tube test as in the present study, that mice at the highest rank began to exhibit depressive-like behaviors only after experiencing repeated “forced losses” (ten forced losses per day for four consecutive days), using an apparatus designed to prevent them from winning. Importantly, they also reported that such depressive-like behaviors did not appear following “natural losses,” as in our tube-test paradigm [67].

In the present study, win–loss outcomes were determined naturally, without constraint, and the mice were always free to escape from the tube (by retreating) and return to the arena. This design minimized social conflict. In addition, we did not observe aggressive or violent behaviors that could harm opponent mice. It is therefore likely that the hierarchy formed among cage mates in the present study was much less stressful for individual mice. Taken together, these observations are against the interpretation that subordinate mice were in a depressive state as a consequence of the defeat experience itself. The neural mechanisms linking the behavioral trait and metabolic regulation of the lowest-rank mice would be an interesting subject of future study.

Another interesting observation of this study, which is the first to describe this phenomenon, is the change in blood glucose regulation induced by inhibition of neuronal excitation in the amygdala area.

Two possible ways of interpreting this result can be considered. First, as this intervention markedly perturbed the rank of each mouse, this change in the social hierarchy affected the behavioral trait of each mouse indirectly or directly through various mechanisms and subsequently affected sympathetic/parasympathetic balance or hypothalamic activities and modified glucose metabolism [7]. Second, as the BLA is involved in regulating various physiological functions, suppression of its neuronal excitability could directly alter autonomic nervous system activities underlying the glucose metabolisms independently of social hierarchy. For example, BLA neurons send direct excitatory projections to the CeA [68], which in turn send inhibitory projections to the dorsal motor nucleus of the vagus nerve [69], a nucleus composed of parasympathetic preganglionic neurons [70]. Anyway, these networks underlying autonomic regulations and social behaviors are mutually interacting each other at various levels of the neuraxis, it might not be a single pathway that leads the BLA suppression to its consequences, i.e., hierarchical changes and glucose metabolism (however, it should be noted that a stimulation of the CeA, but not that of the BLA, triggers pancreatic exocrine in anesthetized rats [71]). As recent advances in dissecting the central mechanism of social hierarchy in mice have identified several brain nuclei underlying social rank regulation [21], [52], isolated manipulation of these structures, such as chemogenetic inhibition of other structures, would help us to understand and elucidate the connections among social hierarchy, diet, and glucose regulation.

### Limitations of this study

There are several limitations to the present study. First, all experiments were conducted exclusively in male mice. Because sex differences have been reported in both social behaviors and metabolic regulation [72], the generalizability of the present findings to female mice remains uncertain.

Second, we assessed social hierarchy solely using the tube test, which is widely used and is considered the most reliable method for determining the rank of co-housed mice [18], [20]. However, it is an indirect proxy for dominance relationships among co-housed mice. We did not incorporate additional behavioral paradigms such as the warm spot test, food competition assays, territorial aggression tests, or home-cage automated tracking, which could provide complementary and potentially more ethologically relevant information about social ranking.

Third, we injected CNO only to rank_control_4 mice and other rank mice did not receive injections to avoid complications arising from injection maneuvers. As one cannot predict the rank of each mouse before a period necessary for sufficient expression of the AAV-transfected genes and it was necessary to inject hM4Di transfection vectors to all mice and rank-dependent intervention was made by selecting the rank_pre-CNO_4 mice. Therefore, the effect of vehicle injection and also the injection maneuver itself on the rank and glucose metabolism should be separately evaluated in the future studies. In addition, although we used AAV5-CaMKIIa vectors for the expression of hM4Di and EGFP to target the BLA neurons, recent studies indicate that subsets of CeA and medial amygdala neurons show CaMKIIa-driven expression of exogenous genes, implying that these regions that are shown to play roles in glycaemic regulation [73], [74] are partly involved in the present chemogenetic suppression of BLA neuron activities. It would be necessary in future studies to limit the expression to the neurons functionally involved in the central regulation of the social rank using appropriate technique [46].

### Clinical implications

The findings of this study have several clinical implications. These findings provide biological evidence that social status, such as position in the social hierarchy, can affect glucose metabolism in a diet-associated manner and may represent a risk factor for diabetes.

Epidemiological studies implicate depression, chronic work stress, and early-life adversity as risk factors for type 2 diabetes [9], [10], [75]. One possible mechanism underlying the association between social class and the prevalence of type 2 diabetes is stress-induced autonomic dysfunction, particularly sympathetic/parasympathetic imbalance [76], [77], [78]. The stress of having low social status reduces parasympathetic activity, which in turn exacerbates insulin resistance [79]. However, in the present study, rank 4 mice presented characteristics of parasympathetic-like predominance. A possible interpretation is that they might have strategically chosen not to win in tube-test matches in order to avoid hierarchy-based conflict and stress [52]. In this sense, the tube test might be used to evaluate not only the social dominance but also the survival strategy of each mouse belonging to a different hierarchy.

## Conclusion

Our finding that the experimental manipulation of central neuronal activity affects glucose metabolism may help to clarify the biological mechanisms underlying the social status-related risk factors for type 2 diabetes and contribute to the development of appropriate individual trait-based therapeutic strategies.

## List of abbreviations

BLA: basolateral amygdala

KD: Kendall distance

GTT: glucose tolerance test

ITT: insulin tolerance test

STT: saline tolerance test

H&E: hematoxylin and eosin

CNO: clozapine-N-oxide

DREADD: designer receptors exclusively activated by designer drugs

CAN: central autonomic network

dmPFC: dorsomedial prefrontal cortex

DRN: dorsal raphe nucleus

PAG: periaqueductal gray

CeA: central amygdala

AgRP: agouti-related protein

## CRediT authorship contribution statement

**Momoyo Ibukuro:** Investigation. **Rikako Ukichi:** Writing – original draft, Investigation, Formal analysis, Data curation. **Fusao Kato:** Writing – review & editing, Writing – original draft, Supervision, Project administration, Methodology, Formal analysis, Conceptualization. **Rimei Nishimura:** Writing – review & editing. **Keiichiro Matoba:** Writing – review & editing. **Yae K Sugimura:** Writing – review & editing. **Yukari Takahashi:** Writing – review & editing, Methodology, Investigation, Data curation, Conceptualization.

## Ethics approval and consent to participate

The manipulation of the animals was approved by the Institutional Animal Care and Use Committee of Jikei University (No. 2018--017, 2019--001, 2019--007) and conformed to the Guidelines for Proper Conduct of Animal Experiments of the Science Council of Japan [25] and the guidelines of the International Association for the Study of Pain [26].

During the preparation of this manuscript, the authors utilized Grammarly and ChatGPT to increase the quality and readability of the English text. The final manuscript was professionally edited for English language by ThinkSCIENCE. The authors have reviewed and edited the content as necessary and take full responsibility for the final version of the publication.

## Funding

This work was partly supported by the MEXT-Supported Program for the Strategic Research Foundation at Private Universities (S1311009) to F.K.

## Declaration of Competing Interest

The authors declare that they have no known competing financial interests or personal relationships that could have appeared to influence the work reported in this paper.

## Data Availability

The datasets used and analyzed in the present study are available from the corresponding author upon reasonable request.
