# Supplementary material for: Modulation of the association between blood glucose homeostasis and social hierarchy among co-housed mice by diet and amygdala activities
Source: J Physiol Sci. 2026 Jan 20;76(1):100058. doi: 10.1016/j.jphyss.2026.100058 (PMC12870758; doi:10.1016/j.jphyss.2026.100058)
Supplement: Supplementary file 1 — Supplementary material [file mmc1.pdf]

Modulation of the association between blood glucose homeostasis and social hierarchy among co-housed mice by diet and amygdala activities

Rikako Ukichi[1,2,a], Yukari Takahashi[1,a], Momoyo Ibukuro[1],Yae K Sugimura[1], Keiichiro Matoba[2], Rimei Nishimura[2], and Fusao Kato[1*]

[1]Department of Neuroscience, [2]Division of Diabetes, Metabolism and Endocrinology, Department of Internal Medicine, The Jikei University School of Medicine, Minato-ku, Tokyo 105-8461, Japan

Supplementary Information

(Figures S1-S5)

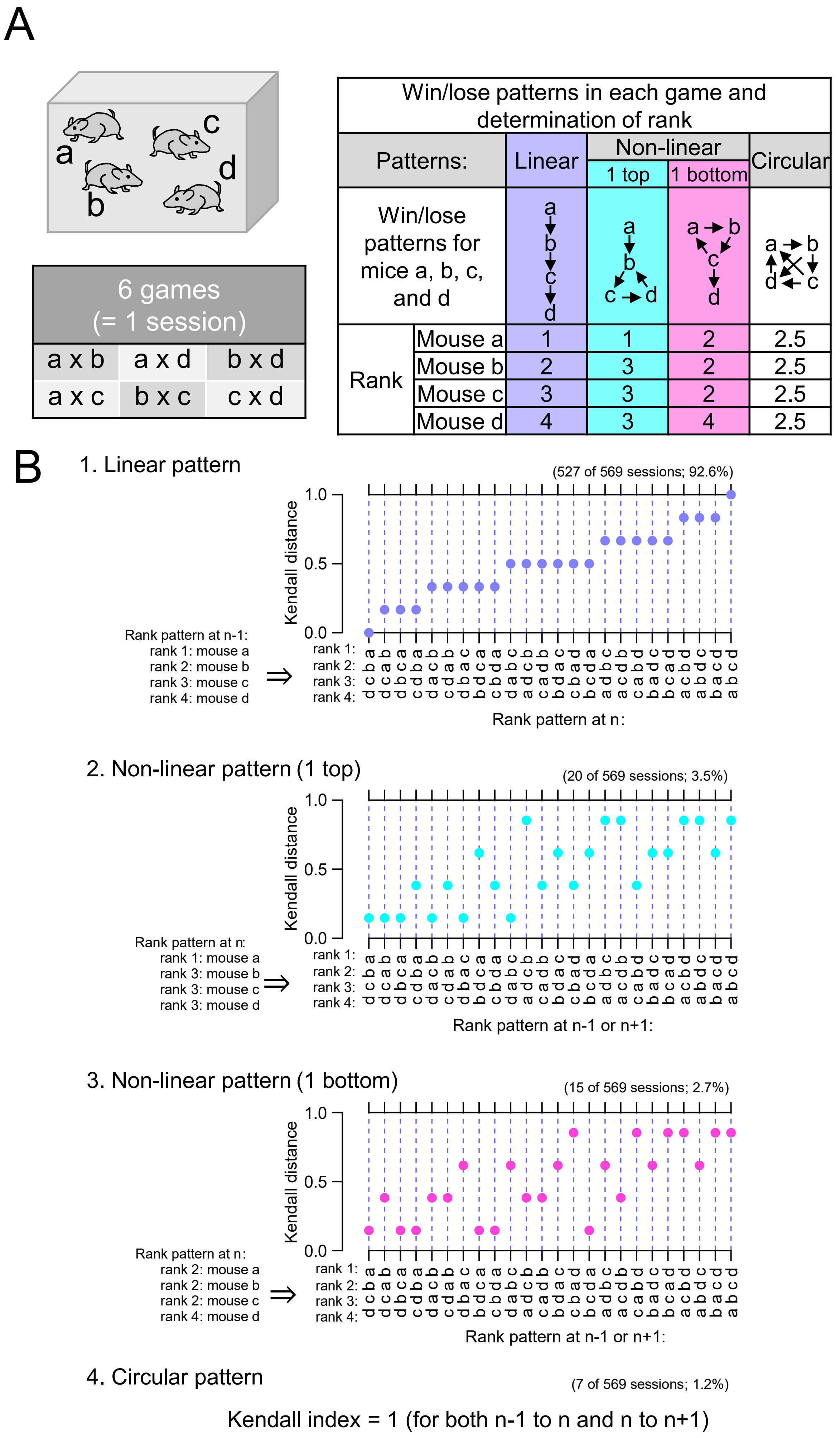
A
a
b
c
d
6 games
(= 1 session)
a x b
a x d
b x d
a x c
b x c
c x d
Win/lose patterns in each game and determination of rank
Patterns:
Linear
Non-linear
1 top
1 bottom
Circular
Win/lose patterns for mice a, b, c, and d
Rank
Mouse a 1 1 2 2.5
Mouse b 2 3 2 2.5
Mouse c 3 3 2 2.5
Mouse d 4 3 4 2.5
B
1. Linear pattern
(527 of 569 sessions; 92.6%)
Kendall distance
1.0
0.5
0.0
Rank pattern at n-1:
rank 1: mouse a
rank 2: mouse b
rank 3: mouse c
rank 4: mouse d
rank 1:
rank 2:
rank 3:
rank 4:
Rank pattern at n:
2. Non-linear pattern (1 top)
(20 of 569 sessions; 3.5%)
Kendall distance
Rank pattern at n:
rank 1: mouse a
rank 3: mouse b
rank 3: mouse c
rank 3: mouse d
Rank pattern at n-1 or n+1:
3. Non-linear pattern (1 bottom)
(15 of 569 sessions; 2.7%)
Kendall distance
Rank pattern at n:
rank 2: mouse a
rank 2: mouse b
rank 2: mouse c
rank 4: mouse d
Rank pattern at n-1 or n+1:
4. Circular pattern
(7 of 569 sessions; 1.2%)
Kendall index = 1 (for both n-1 to n and n to n+1)

Supplementary figure 1. Protocols for rank assessment and Kendall distance calculation

A. Rank assessment by the tube test. The hierarchical order among four co-housed mice (a, b, c, d) was determined using a round-robin tube test consisting of six pairwise games per session. The mouse with the highest winning percentage was assigned rank 1 (most dominant), and the mouse with the lowest winning percentage was assigned rank 4 (least dominant). Rank determination was based on the outcomes of all six games (= 1 session). Representative win/lose patterns and the corresponding rank assignments are summarized in the table.

B. Calculation of the Kendall distance (KD). KD quantifies the degree of dissimilarity between two rank orders and is defined as the minimum number of pairwise swaps required to make them identical. B1: KD values for linear rank patterns. B2: KD values for non-linear patterns with a single top-ranked mouse. B3: KD values for non-linear patterns with a single bottom-ranked mouse. B4: KD values for circular patterns. KD equals 1 for both transitions from any pattern to a circular pattern and from a circular pattern to any other pattern. Among the 569 consecutive sessions analyzed in this study, the probabilities of observing each KD pattern were 92.6% (linear), 3.5% (non-linear, top), 2.7% (non-linear, bottom), and 1.2% (circular).

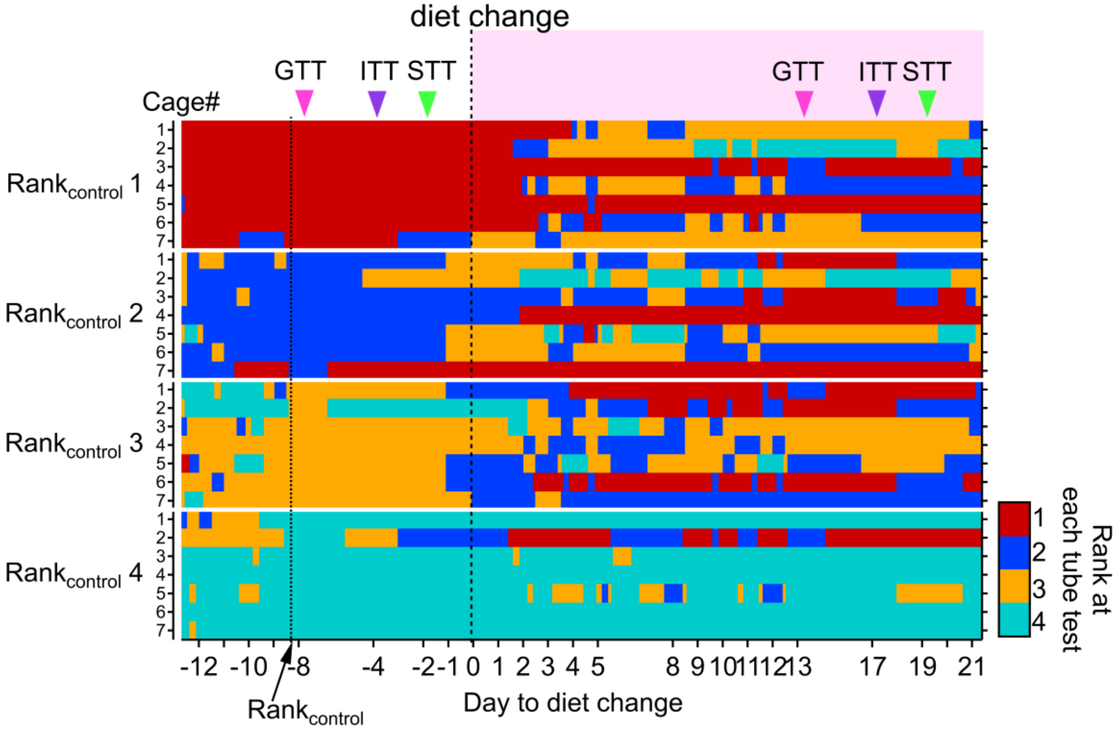
diet change
GTT
ITT
STT
GTT
ITT
STT
Cage#
Rankcontrol 1
Rankcontrol 2
Rankcontrol 3
Rankcontrol 4
Rank at each tube test
-12 -10 -8 -4 -2 -1 0 1 2 3 4 5 8 9 10 11 12 13 17 19 21
Rankcontrol
Day to diet change

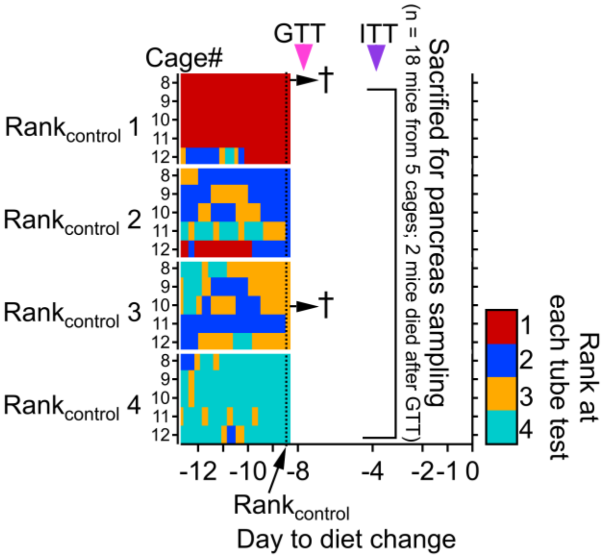
GTT
ITT
Cage#
Rankcontrol 1
Rankcontrol 2
Rankcontrol 3
Rankcontrol 4
Sacrified for pancreas sampling
(n = 18 mice from 5 cages; 2 mice died after GTT)
Rank at each tube test
-12 -10 -8 -4 -2 -1 0
Rankcontrol
Day to diet change

Supplementary figure 2. Temporal dynamics of social rank before and after the dietary intervention.

Color-coded heatmap showing daily rank transitions of individual mice (rows) within each $rank_{control}$ across seven cages. $Rank_{control}$4 mice showed relatively stable social positions throughout the observation period. The top and bottom panels show heatmaps of mice without and with for pancreatic sample collection, respectively. In the bottom panel, mice from 5 cages (cage# 8-12) were sacrificed immediately after the ITT for pancreas sampling. This resulted in pancreatic sampling from 18 mice as two mice died during the GTT (†).

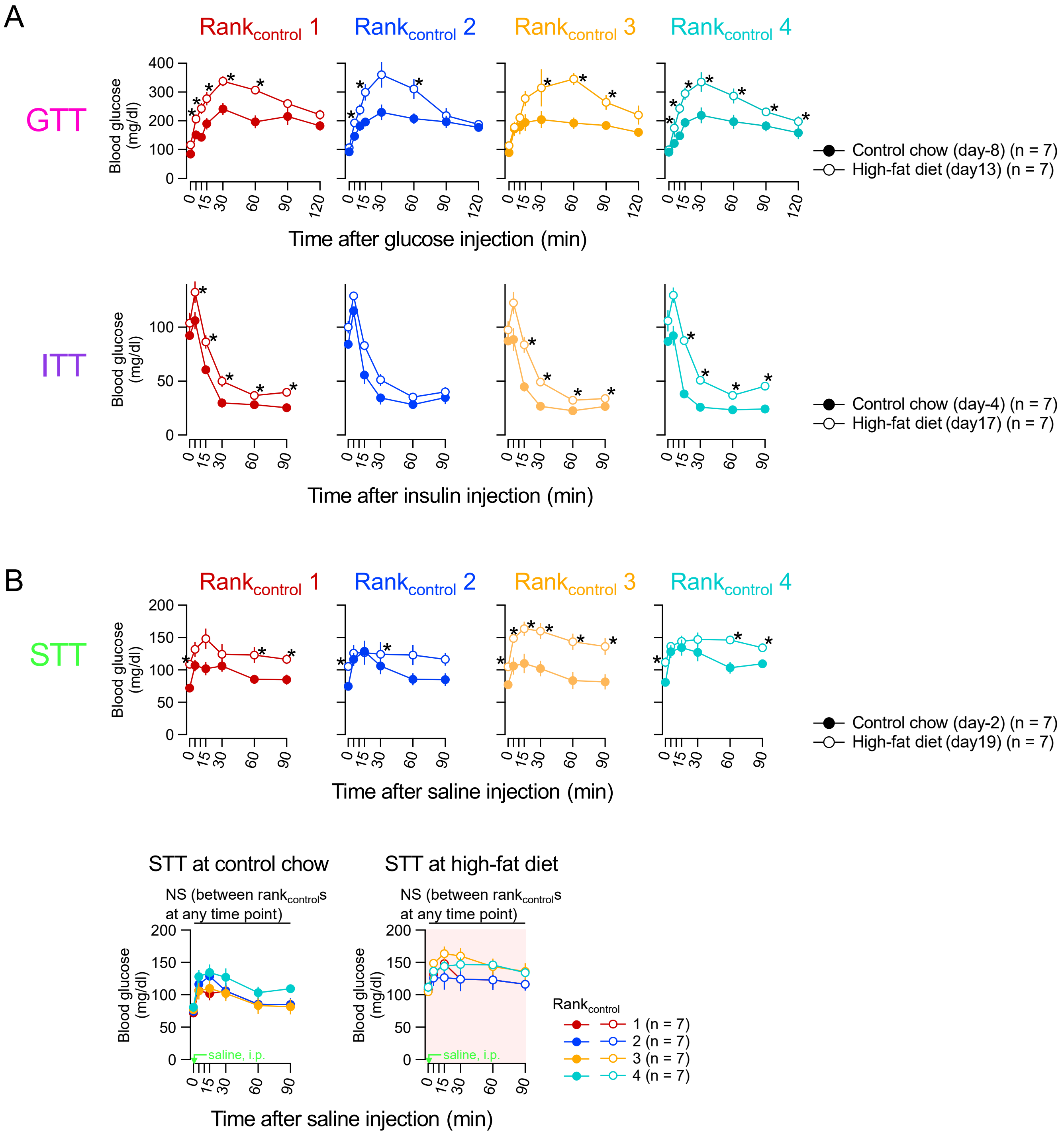
A
GTT
ITT
Rankcontrol 1
Rankcontrol 2
Rankcontrol 3
Rankcontrol 4
Blood glucose (mg/dl)
Time after glucose injection (min)
Control chow (day-8) (n = 7)
High-fat diet (day13) (n = 7)
Time after insulin injection (min)
Control chow (day-4) (n = 7)
High-fat diet (day17) (n = 7)
B
STT
Time after saline injection (min)
Control chow (day-2) (n = 7)
High-fat diet (day19) (n = 7)
STT at control chow
STT at high-fat diet
NS (between rankcontrols at any time point)
saline, i.p.
Rankcontrol
1 (n = 7)
2 (n = 7)
3 (n = 7)
4 (n = 7)

Supplementary figure 3. Comparison of glucose metabolism-associated parameters under control chow and high-fat diet conditions in the same cohort.

A. Time courses of the average blood glucose concentrations in each $rank_{control}$ at GTT (top graphs) and ITT (bottom graphs). Colored lines and filled and open circles indicate the blood glucose concentrations under control chow and high-fat diet conditions, respectively. For the statistical comparisons of the blood glucose concentrations between diet conditions, paired *t*-tests were performed at each time point after glucose injection (for GTTs) and insulin injection (for ITTs). *, $p < 0.05$. Mean ± SEM.

B. Time courses of blood glucose concentrations in STTs. The top graphs show comparisons before and after a diet change in mice of different $rank_{control}$ (paired t-test, *, $p < 0.05$; Mean ± SEM). In the bottom graphs, colored lines and circles represent mean values by $rank_{control}$ (mean ± SEM, n = 7 for all $rank_{control}$). No significant differences were detected at any time point (one-way ANOVA).

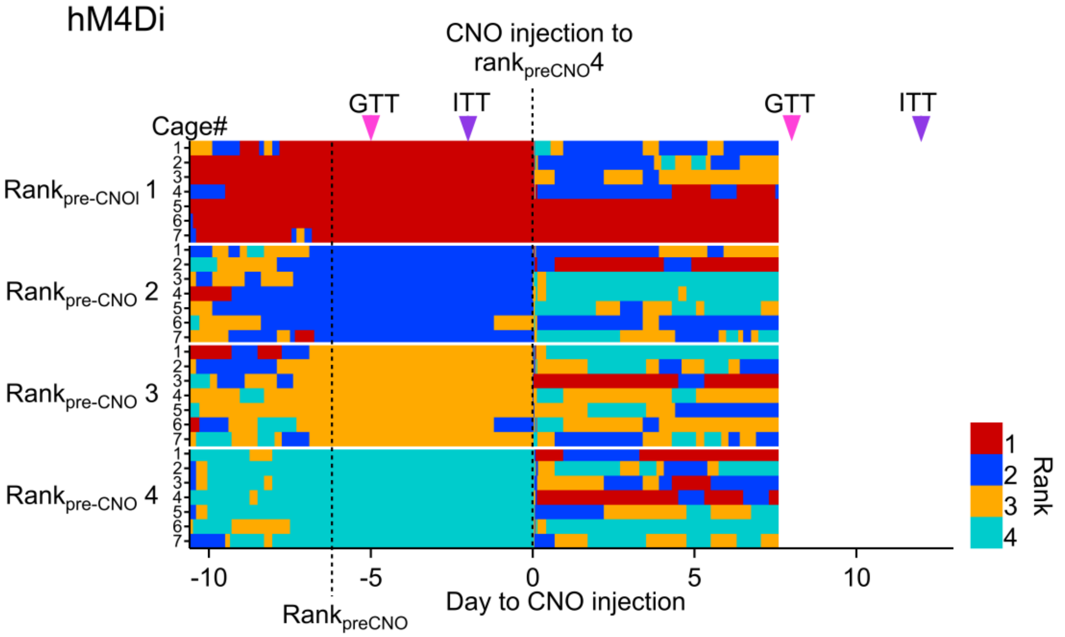
hM4Di
CNO injection to rankpreCNO4
GTT
ITT
GTT
ITT
Cage#
Rankpre-CNOl 1
Rankpre-CNO 2
Rankpre-CNO 3
Rankpre-CNO 4
-10
-5
0
5
10
Day to CNO injection
RankpreCNO
Rank
1
2
3
4

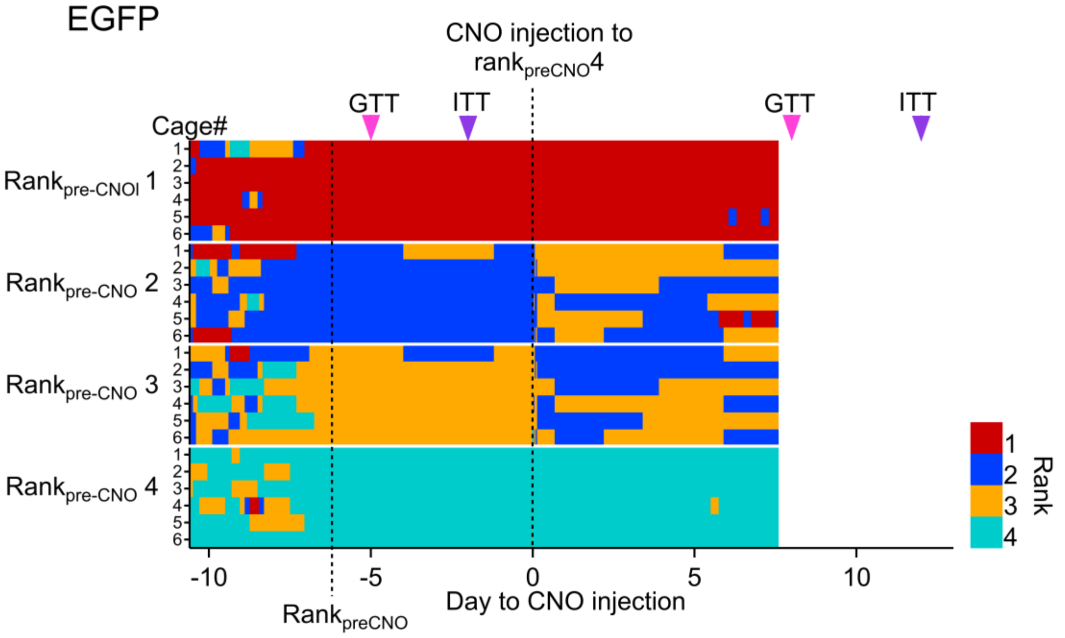
EGFP
CNO injection to rankpreCNO4
GTT
ITT
GTT
ITT
Cage#
Rankpre-CNOl 1
Rankpre-CNO 2
Rankpre-CNO 3
Rankpre-CNO 4
-10
-5
0
5
10
Day to CNO injection
RankpreCNO
Rank
1
2
3
4

Supplementary figure 4. Temporal dynamics of social rank before and after chemogenetic inhibition in the basolateral amygdala neurons of rank 4 mice.

Color-coded heatmap showing the daily rank transitions of individual mice (rows) within each $rank_{pre\text{-}CNO}$ across seven cages in the hM4Di group and six cages in the EGFP group.

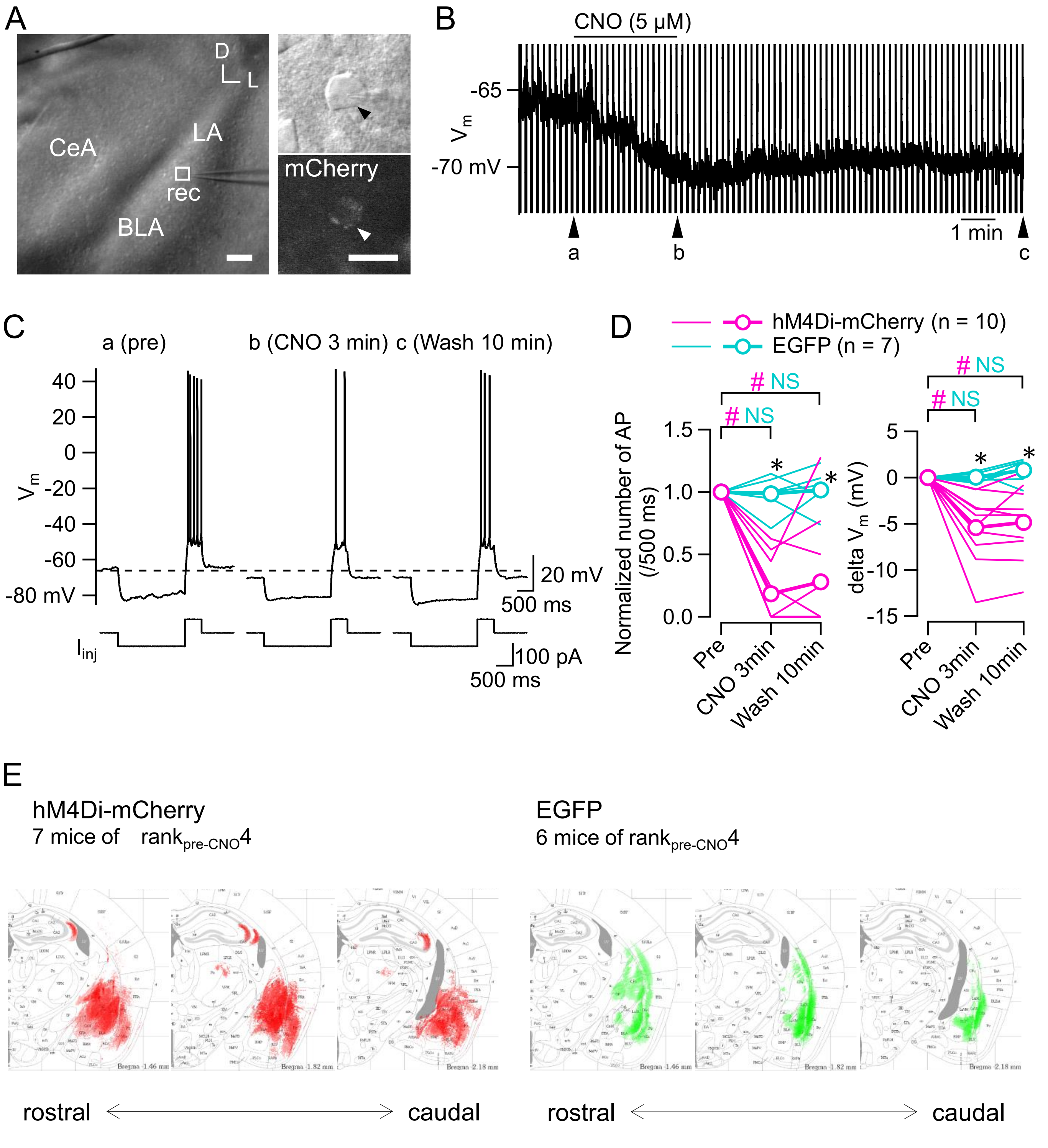
A
D
L
CeA
LA
rec
BLA
mCherry
B
CNO (5 μM)
-65
-70 mV
Vm
1 min
a
b
c
C
a (pre)
b (CNO 3 min) c (Wash 10 min)
40
20
0
-20
-40
-60
-80 mV
Vm
20 mV
500 ms
Iinj
100 pA
500 ms
D
hM4Di-mCherry (n = 10)
EGFP (n = 7)
NS
Normalized number of AP (/500 ms)
1.5
1.0
0.5
0.0
Pre
CNO 3min
Wash 10min
delta Vm (mV)
5
0
-5
-10
-15
E
hM4Di-mCherry
7 mice of rankpre-CNO4
EGFP
6 mice of rankpre-CNO4
Bregma -1.46 mm
Bregma -1.82 mm
Bregma -2.18 mm
rostral
caudal

Supplementary figure 5. Electrophysiological confirmation of the effect of CNO on BLA neurons expressing hM4Di.

A–D. A representative electrophysiological recording of the membrane potential of an hM4Di-mCherry-expressing LA/BLA neuron.

A. A representative image with oblique illumination from a coronal slice used for whole-cell patch-clamp recording from LA/BLA neurons. BLA, basolateral amygdala; LA, lateral amygdala; CeA, central amygdala; rec, recording pipette. D and L, dorsal and lateral directions. The right panels are magnified images of the area indicated by a rectangle in the left panel. The arrowheads indicate an hM4Di-mCherry-positive neuron that was recorded. Scale bars are 100 μm (for the left panel) and 20 μm (for the right upper and lower panels).

B. The effect of CNO (5 μM) applied into the recording chamber on the resting membrane potential of hM4Di-mCherry-positive cells. Representative trace of a patch-clamp recording in current clamp mode from the neuron expressing hM4Di-mCherry shown in A. The regularly repeated vertical shifts represent parts of passive membrane responses to hyperpolarizing and depolarizing currents injected every 10 s.

C. Representative responses of the membrane potential before CNO application (a in B, pre), 3 min after CNO application (b in B, CNO 3 min), and 10 min after the start of CNO washout (c in B, Wash 10 min). The rectangular hyperpolarizing pulse (2 s, −60 pA) followed by a depolarizing pulse (500 ms, 60 pA) was injected every 10 s. The dashed line is the average resting membrane potential recorded every 10 s for 1 min before CNO application.

D. A summary of the effect of CNO on the number of action potentials induced by a depolarizing pulse and the resting membrane potential recorded from hM4Di-mCherry-positive neurons (pink line and circles, 10 neurons from 3 mice) and EGFP-positive control neurons (light blue line and circles, 7 neurons from 2 mice). The left graph shows the number of action potentials (APs) normalized to that under ACSF (Pre). The right graph shows the difference in the membrane potential (V_m) normalized to that under ACSF (Pre). Thick lines and circles represent the average in each group. CNO (5 μM) hyperpolarized the membrane potential (right) and decreased the number of action potentials (left) in hM4Di-mCherry-positive neurons; Mann–Whitney *U* test; ***p* < 0.01, ACSF vs CNO; Wilcoxon signed-rank test.

E. A summary of the fluorescence distribution of hM4Di-mCherry (left, 1 slice/mouse, 7 mice) and EGFP (right, 1 slice/mouse, 6 mice) near the AAV injection

site in the right BLA of representative coronal slices. The fluorescence distribution reflects not only the somata of AAV-infected neurons but also their neuronal processes, including axons and dendrites, and therefore does not necessarily indicate the precise localization of the injection site.

Supplementary Information (related to Figure S5)

We prepared brain slices for electrophysiological recordings according to previously described procedures from our laboratory [44]. The mice were first transcardially perfused with an ice-cold cutting solution under isoflurane anesthesia, and the brain was removed. A block of the forebrain containing the amygdala was dissected out and cut at the midline in the ice-cold cutting solution composed of (in mM) 2.5 KCl, 0.5 $CaCl_2$, 10 $MgSO_4$, 1.25 NaH_2PO_4, 2 thiourea, 3 sodium pyruvate, 93 N-methyl-D-glucamine, 20 HEPES, 12 N-acetyl-L-cysteine, 25 D-glucose, 5 L-ascorbic acid, and 30 $NaHCO_3$ equilibrated with 95% O_2 + 5% CO_2 (osmolarity, ~290 mOsm/kg; the pH of the solution was titrated to 7.1–7.5 with concentrated HCl). The dissected hemisphere containing the amygdala was secured on the cutting stage of a vibrating blade slicer (Neo Linear Slicer MT; Dosaka EM, Kyoto, Japan). Brain slices of 300-mm thickness were prepared. The slices were first incubated in a holding chamber at 34°C for 15 min. Then, the slices were transferred to another holding chamber containing artificial cerebrospinal fluid (ACSF) composed of (in mM) 119 NaCl, 2.5 KCl, 2 $CaCl_2$, 2 $MgSO_4$, 1.25 NaH_2PO_4, 12.5 D-glucose, 5 L-ascorbic acid, 2 thiourea, 3 sodium pyruvate, and 26 $NaHCO_3$ (pH ~7.3, bubbled with 95% O_2 and 15% CO_2; osmolality, approximately 300–310 mOsm/kg H_2O) at room temperature (20–25°C). Each slice was transferred to a recording chamber (volume, approximately 0.4 mL) and fixed with nylon grids attached to a platinum frame. The slice was submerged in the chamber and superfused continuously at a rate of 1.8 to 3 mL/min with the ACSF described above.

BLA neurons were identified visually under an upright microscope (BX-51WI; Olympus, Tokyo, Japan) with oblique illumination. Images from living slices during electrophysiological recordings were captured using a CCD camera (IR-1000; DAGE-MTI, Michigan City, IN) and stored digitally on a computer. Patch-clamp electrodes were made from borosilicate glass pipettes (1B120F-4; World Precision Instruments, Sarasota, FL). The tip resistance of the electrode was 5 to 8 MΩ. The composition of the internal solution was (in mM) 120 potassium gluconate, 6 NaCl, 1 $CaCl_2$, 2 $MgCl_2$, 2 ATP Mg, 0.5 GTP Na, 12 phosphocreatine Na_2, 5 EGTA, and 10 HEPES hemisodium (pH 7.3, as adjusted with KOH; osmolarity, ~290 mOsm/kg). Whole-cell membrane potentials of transduced neurons (identified by mCherry or EGFP fluorescence) were recorded using an Axopatch 700B amplifier (Molecular Devices, San Jose, CA), filtered at 2 kHz, and digitized at 10 kHz with 16-bit resolution using a PowerLab interface (AD Instruments, Dunedin, New Zealand). Input resistance,

resting membrane potential, and whole-cell capacitance were measured immediately after the establishment of whole-cell mode by membrane rupture. The resting membrane potential was recorded (in current clamp mode) in normal ACSF for 5 min before the addition of 5 μM CNO (Enzo Life Sciences Inc., Farmingdale, NY) for 3 min. Stock solutions of CNO were dissolved in water, kept frozen at −30°C, and then dissolved in ACSF to their final concentration on the day of the experiment. A rectangular hyperpolarizing pulse (100–300 ms, −20 pA) followed by a depolarizing pulse (500 ms, 100–120 pA) was injected every 10 s to observe the electroresponsive properties of the neurons. All recordings were made at room temperature (20–25°C). Oblique illumination and epifluorescence images were captured using the same camera and were overlaid in ImageJ (ver. 1.48; National Institutes of Health, Bethesda, MD) with modification of the brightness and contrast alone.
